# Bayesian hierarchical vector autoregressive models for patient-level predictive modeling

**DOI:** 10.1371/journal.pone.0208082

**Published:** 2018-12-14

**Authors:** Feihan Lu, Yao Zheng, Harrington Cleveland, Chris Burton, David Madigan

**Affiliations:** 1 Department of Statistics, Columbia University, New York, NY, United States of America; 2 Department of Psychology, University of Alberta, Edmonton, AB, Canada; 3 Human Development and Family Studies, The Pennsylvania State University, University Park, PA, United States of America; 4 Academic Unit of Primary Medical Care, The University Of Sheffield, Sheffield, United Kingdom; Universitatsmedizin Greifswald, GERMANY

## Abstract

Predicting health outcomes from longitudinal health histories is of central importance to healthcare. Observational healthcare databases such as patient diary databases provide a rich resource for patient-level predictive modeling. In this paper, we propose a Bayesian hierarchical vector autoregressive (VAR) model to predict medical and psychological conditions using multivariate time series data. Compared to the existing patient-specific predictive VAR models, our model demonstrated higher accuracy in predicting future observations in terms of both point and interval estimates due to the pooling effect of the hierarchical model specification. In addition, by adopting an elastic-net prior, our model offers greater interpretability about the associations between variables of interest on both the population level and the patient level, as well as between-patient heterogeneity. We apply the model to two examples: 1) predicting substance use craving, negative affect and tobacco use among college students, and 2) predicting functional somatic symptoms and psychological discomforts.

## 1 Introduction

Analyses of patient-level observational healthcare databases underpin much of the current evidence base for healthcare practice. Administrative claims, electronic health record (EHR), and patient diary databases in particular have seen increased use in the past decade owing to greater availability at lower costs and technological advances that made computational processing on large-scale data more feasible [1]. Because the data reflect healthcare activity within a real-world population, they offer the potential to complement clinical trial results. Administrative claims databases have been the most actively used observational healthcare data source. These databases typically capture data elements used within the reimbursement process, as providers of healthcare services (e.g., physicians, pharmacies, hospitals, and laboratories) must submit encounter information to enable payment for their services [2].

Neither administrative claims nor EHRs represent the ideal information required to generate reliable evidence. For example, diagnoses recorded on medical claims are used to support justification for the payment for a given visit or procedure and “up-coding” may occur, or, a given diagnosis could represent the condition that the procedure was used to rule out or could be an administrative artifact. Some diagnosis codes have been studied through source record verification and have demonstrated impressive performance characteristics (see, for example, [3], or [4]), whereas other conditions and systems provide less certainty (see, for example, [5], [6], or [7]). Limitations exist in EHR systems as well, in which, apart from concerns about incomplete capture, data may be artificially manipulated to serve clinical care (e.g., an incorrect diagnosis recorded to justify a desired medical procedure).

Patient diary databases capture fewer variables and smaller numbers of patients but offer significant advantages. In particular, diaries can capture longitudinal data about activities of daily living, mood, and personal habits and not just when a patient is seeking healthcare. Patient diaries support significant research activities in chronic disease management [8], [9], [10]. Electronic patient diaries capture data via a web interface or a phone or tablet and represent the most common modality. Participating patients make diary entries either daily or at multiple times per day, or at set intervals.

Major use cases for observational healthcare databases include inferences about the causal effects of healthcare interventions (typically via cohort, case-control, or self-controlled case series designs), healthcare characterization (see, for example, [11]), and patient-level predictive modeling (PLPM), the primary focus of this paper. PLPM lies right at the core of healthcare practice and so-called precision medicine. At least implicitly, every healthcare intervention decision involves a patient-level prediction.

Many researchers have considered the problem of developing predictive models from patient-level data (see, for example, [12], [13]). In the vast majority of cases, researchers generated a set of candidate covariates thought to be related to the outcome of interest and employed traditional statistical or machine learning algorithms to predict the outcome using these covariates. The authors of He et al. (2014), for example, predict hospital readmissions using logistic regression with a modified forward variable selection algorithm to choose features from the set of all indicator variables for any past occurrence of a medical concept with a diagnostic code [14]. None of the features in their study, however, conveys any information about time. This is the same for a few other studies that utilized similar approaches to predict medical conditions such as asthma [15], cardiovascular diseases [16] and hypertension disorders [17].

Some authors have attempted to incorporate explicit temporal information into patient-level predictive models. In the context of stroke prediction, Shahn et al. (2015) proposed a “relational random forests” (RRF) that capture “motifs” in the medical history that are predictive of specific future events [18]. Motifs capture healthcare episodes such as “received drug X or diagnosis Y then suffered condition Z within T days.” In their target application, RRF showed modest benefits in predictive performance. Wang et al. (2012) consider a patient’s health history as the superposition and concatenation of multiple pattern matrices [19]. Each pattern matrix specifies a rigid temporal relationship among health events that repeats over time. They employ a matrix factorization algorithm to learn the pattern matrices, which can then be used to construct features for predictive models. Brzan et al (2017) show that incorporation of historical patient data improves predictive performance for hospital readmission prediction [20].

In this paper, we approach PLPM as a multivariate time series modeling problem and consider a temporal generative model for the medical record. We adopt a Bayesian approach and consider applications to patient diary data in the context of drug and alcohol treatment and functional somatic symptoms. We explore the predictive performance of this model. Since the model offers advantages in terms of interpretability as compared with alternatives such as random forests and deep learning, we explore insights derived from the model.

## 2 Vector autoregressive models for patient data

Vector autoregressive (VAR) models represent a mainstay of multivariate time analysis [21]. Previous studies have applied VAR models to patient diary data (see, for example, [22] and [23]). However, these studies fit separate models for each individual patient and have a few limitations. The patient-specific VAR coefficient estimates, for example, could have large variance (or even spurious estimates) when the number of observations for a particular patient is small relative to the number of parameters in the coefficient matrices, leading to potentially high variance in future data prediction. Further, to analyze between-subject heterogeneity, existing methods utilize ad-hoc approaches such as clustering analysis on the patient-specific VAR coefficient estimates [22]. This is, however, statistically sub-optimal due to loss of information by using coefficient estimates rather than the original data. Moreover, no quantification of the between-subject heterogeneity is provided.

Here we take a Bayesian approach and simultaneously model *all* patients using a hierarchical model. Bayesian VAR models have been widely applied in various fields of study such as economics [24], [25], [26], neuroimaging [27] and more recently psychology [28]. We adopt a flexible elastic-net prior to induce sparsity in the estimation of the coefficients and to aid interpretability [29]. Specifically, this prior is able to select the most predictive variables in the model and remove unimportant ones, leading to a model with a smaller number of important variables and thus higher interpretability. The hierarchical structure for the subject-level coefficients enables simultaneous inference about both the population-level and the subject-level coefficients and, in our target application, improves predictive performance. Our approach also enables estimation of between-subject heterogeneity in a quantitative way by contrast with the current clustering analysis, leading to higher interpretability of these parameters.

### 2.1 Sparse hierarchical VAR model

Let *N* denote the number of patients. We assume that each patient records diary measurements at *T*_*n*_ time points, *n* = 1, …, *N* on *R* variables. In what follows we assume daily measurements. Let ***y***_*nt*_ = (*y*_*nt*1_, …, *y*_*ntR*_)^*T*^ be a column vector representing the measurements at time *t*, *t* = 1, …, *T*_*n*_, for patient *n*, *n* = 1, …, *N*. Then, for each patient, the sparse hierarchical VAR(*p*) model assumes

$$\begin{array}{c} y_{nt}=\sum_{i=1}^{p}A_{ni}y_{n,t-i}+\epsilon_{nt},t=p+1,\ldots ,T_{n} \end{array}$$ (1)

where *A*_*ni*_, *i* = 1, …, *p* is a *R* × *R* coefficient matrix representing the lag-*i* association, *p* is the model order, i.e., the total number of past time steps used as predictors, and ***ϵ***_*nt*_ is the error term assumed to follow i.i.d. multivariate Normal (MVN) distribution with mean **0** and precision matrix Λ: ***ϵ***_*nt*_ ∼ MVN(**0**, Λ^−1^). We write the model in concise matrix form as:

$$\begin{array}{c} y_{n}=(H_{n}^{T}⊗I_{B})w_{n}+\epsilon_{n} \end{array}$$ (2)

where

$$\begin{array}{c} y_{n}=(\begin{array}{c} y_{n,p+1}\\ \vdots \\ y_{nT_{n}} \end{array}) \end{array}$$ (3)

is the response variable and ⊗ is the Kronecker product,

$$\begin{array}{c} H_{n}=(\begin{array}{ccc} y_{np} & \cdots & y_{n,T_{n}-1}\\ \vdots & \ddots & \vdots \\ y_{n1} & \cdots & y_{n,T_{n}-p} \end{array}) \end{array}$$ (4)

represents the regressors,

$$\begin{array}{c} w_{n}=vec\left(\left[A_{n1},\ldots ,A_{np}\right]\right) \end{array}$$ (5)

is the vectorized coefficients of length *R*^2^ *p*,

$$\begin{array}{c} \epsilon_{n}=(\begin{array}{c} \epsilon_{n,p+1}\\ \vdots \\ \epsilon_{nT_{n}} \end{array})∼\text{MVN}\left(0,I_{T_{n}-p}⊗\Lambda^{-1}\right) \end{array}$$ (6)

is the stacked error term, and *I*_*k*_ represents a *k* × *k* identity matrix.

Here we assume that the precision matrix Λ is the same for all patients but the coefficient vector ***w***_*n*_ is unique for patient *n*. Specifically, we assume that the coefficient vector for the *n*-th patient comprises two parts:

$$\begin{array}{c} w_{n}=w+v_{n} \end{array}$$ (7)

where ***w***, is the population-level coefficient and is the same across all patients, ***v***_*n*_ is the patient-level coefficient for the *n*-th patient.

For the population-level coefficient, we adopt the doubly adaptive elastic-net prior [29]. Specifically, we assume that the prior for ***w*** follows a multivariate Normal distribution with mean **0** and precision matrix *D*:

$$\begin{array}{c} w|D∼\text{MVN}(0,D^{-1}) \end{array}$$ (8)

where *D* is a diagonal matrix and the diagonal elements depend on hyperparameters $2\tau_{k}^{2}$ and λ_2,*k*_ > 0, *k* = 1, …, *R*^2^ *p*:

$$\begin{array}{c} \text{diag}\left(D\right)=(\lambda_{2,1}+\frac{1}{2\tau_{1}^{2}},\ldots ,\lambda_{2,R^{2}p}+\frac{1}{2\tau_{R^{2}p}^{2}}) \end{array}$$ (9)

Here $2\tau_{k}^{2},k=1,\ldots ,R^{2}p$ is assumed to follow independent Exponential distribution with rate parameter $\frac{\lambda_{1,k}^{2}}{2\xi_{k}^{2}}$:

$$\begin{array}{c} 2\tau_{k}^{2}|2\xi_{k}^{2},\lambda_{1,k}∼E(\frac{\lambda_{1,k}^{2}}{2\xi_{k}^{2}}) \end{array}$$ (10)

where λ_1,*k*_ > 0, *k* = 1, …, *B*^2^ *p*, and $\xi_{k}^{2},k=1,\ldots ,R^{2}p$, depends on the error precision matrix Λ through:

$$\begin{array}{c} \xi_{k}^{2}=M_{k,k}-(M_{k,k+1},\ldots ,M_{k,R^{2}p}){\left(\begin{array}{ccc} M_{k+1,k+1} & \cdots & M_{k+1,R^{2}p}\\ \vdots & \ddots & \vdots \\ M_{R^{2}p,k+1} & \cdots & M_{R^{2}p,R^{2}p} \end{array}\right)}^{-1}(\begin{array}{c} M_{k,k+1}\\ \vdots \\ M_{k,R^{2}p} \end{array}) \end{array}$$ (11)

$$\begin{array}{c} M=I_{Rp}⊗\Lambda^{-1}. \end{array}$$ (12)

We also assume that λ_1,*k*_ and λ_2,*k*_, *k* = 1, …, *R*^2^ *p* follow two i.i.d. Gamma distributions:

$$\begin{array}{c} \lambda_{1,k}∼\Gamma \left(\mu_{1},\nu_{1}\right) \end{array}$$ (13)

$$\begin{array}{c} \lambda_{2,k}∼\Gamma \left(\mu_{2},\nu_{2}\right) \end{array}$$ (14)

where *μ*_1_, *ν*_1_, *μ*_2_, *ν*_2_ are the corresponding mean and degree of freedom parameters which are assumed to be known. Here, λ_1,*k*_ and λ_2,*k*_ represent the L1 and L2 tuning parameters for the *k*-th element of ***w***, respectively. It can be shown that the conditional distribution for each of the elements of ***w*** given the error precision matrix Λ comprise a Normal distribution and a Laplace distribution. In other words, this prior generalizes the adaptive Lasso and provides adaptive shrinkage for the elastic-net regularization. Note that conditioning on the error precision matrix Λ is important as it ensures unimodal posterior distribution for each of the elements of ***w*** [30].

For the patient-level coefficients, we apply conjugate i.i.d. multivariate Normal priors with mean **0** and diagonal precision matrix Θ_*v*_:

$$\begin{array}{c} v_{n}|\Theta_{v}∼\text{MVN}(0,\Theta_{v}^{-1}) \end{array}$$ (15)

for *n* = 1, …, *N*, where the diagonal elements of Θ_*v*_ are

$$\begin{array}{c} \text{diag}\left(\Theta_{v}\right)=\left(\theta_{v1},\ldots ,\theta_{v,R^{2}p}\right) \end{array}$$ (16)

for which we apply the Gamma hyperpriors on each of the diagonal elements *θ*_*vk*_, *k* = 1, …, *R*^2^ *p*, i.i.d.:

$$\begin{array}{c} \theta_{vk}∼\Gamma \left(k,s\right) \end{array}$$ (17)

where *k* and *s* are the known mean and degree of freedom parameters (see [29] for parameterization of the Gamma distribution). We assume that the patient-level coefficients are independent of the population-level coefficients given the hyperparameters.

Finally, we use a conjugate Wishart distribution for the error precision matrix Λ:

$$\begin{array}{c} \Lambda ∼\text{Wishart}(K,\nu ) \end{array}$$ (18)

where *K* is the *R* × *R* scale matrix and *ν* is the degree of freedom parameter, both assumed to be known.

### 2.2 Posterior inference

We use Markov Chain Monte Carlo (MCMC) to generate draws from the posterior distribution, specifically, a Gibbs sampler with parameter expansion [31]. A common computational challenge of Gibbs sampling for hierarchical regression models is that the sampler can be very slow when there is high dependence between the coefficients and their variance parameters. For our model, there is strong dependence between ***v***_*n*_ and its precision parameters Θ_*v*_: when the current draw of Θ_*v*_ is large, the next draw of ***v***_*n*_ will be small, which in turn makes the next draw of Θ_*v*_ even larger, and so on, so the sampler can take an impractically long time to explore the entire parameter space.

Using parameter-expansion allows us to reduce inter-parameter correlations and speeds convergence. Specifically, we add an element-wise multiplicative factor ***α*** to ***v***_*n*_ (i.e., multiply *α*_*k*_ to *v*_*nk*_, *k* = 1, …, *R*^2^ *p*) and draw ***α*** just like other parameters. In this case, when the current draw of Θ_*v*_ is large, the next draw of ***v***_*n*_ will be small, but the next draw of ***α*** will be large, which results in the next draw of ***v***_*n*_ in a normal range. Thus, the expanded parameterization allows the Gibbs sampler to move in more directions and avoid getting stuck. These new parameters have no meaning and are not identified in the posterior distribution. However, we are not interested in either ***α*** or ***v***_*n*_. Rather, the subject-level parameters in this new parameterization become $v_{n}^{*}=\alpha *v_{n}$ where * stands for element-wise multiplication (see S1 Appendix for parameter expansion and S2 Appendix for detailed descriptions of the full conditionals used in the posterior sampling).

After obtaining the posterior distributions, we use the posterior modes as the point estimates for the parameters in the model. Using posterior modes as point estimates induces sparsity, and is equivalent to the estimates given by the penalized likelihood methods such as Lasso [30] [32]. Here we use the values with the highest empirical density as the modes of the posterior distribution. This is a crude approach to find the posterior modes but is computationally efficient for problems with many parameters.

Finally, we use AIC as a criterion to select the optimal order of the VAR model.

## 3 Application 1: Predicting substance use craving, negative affect, and tobacco use among young adults in recovery

College and university students experience high rates of substance use disorders, with 22.9% meeting the diagnostic criteria versus 8.5% of the general population (National Center on Addiction and Substance Abuse at Columbia University [CASA], 2007). Recognition of this problem has led at least 20 colleges and universities to develop collegiate recovery communities (CRC) [33] that provide comprehensive recovery support services and one of these communities provided the diary data for the Zheng et al. (2013) study [22]. Day-to-day associations among substance use craving, negative affect (i.e., emotional discomforts such as “stressed,” “upset,” “scared,” “hostile,” and “irritable”), and tobacco use among 30 college students who are smokers and in 12-step recovery from drug and alcohol addictions are analyzed in this study. The 12-step program was first created by Alcoholics Anonymous (www.aa.org) and is a frequently used treatment modality for various types of addictions. The intraindividual variability of relevant psychological states combined with the “one day at a time” nature of sustained abstinence warrants a day-to-day investigation of substance use recovery. Zheng et al. (2013) fit first-order vector autoregression models to each individual predicting daily levels of substance use craving, negative affect and tobacco use.

Extensive research has established the relevance of craving, negative affect, and tobacco use as key criteria in recovery research (e.g., [34]). In turn, negative affect is believed to be a predictor of craving, and tobacco use is both an important correlate of substance use and craving and a serious public health risk in its own right. Whether tobacco or other substance use (e.g., alcohol and drugs) has any beneficial effects for persons in recovery remains an open question. Quantifying the bidirectional associations among these variables serves as a secondary goal of Zheng et al. (2013) study.

Zheng et al. (2013) present a series of conclusions finding overall that the study revealed “substantial person-level heterogeneity in the day-to-day processes that challenge continued abstinence within a college recovery community, providing a picture of accumulated daily recovery risk that could threaten abstinence over both the short and long term.”

### 3.1 Data and preprocessing

The original data in Zheng et al. (2013) comprise 55 adult addict patients from CRC at a Southwestern university. In this study, we included 25 patients from the same sample, excluding 12 of the 55 who are non-smokers, 4 whose diary records exhibit no day-to-day variance, and 14 who have 3 or more missing daily records. All participants were fully anonymized before accessing the data. All are non-Hispanic White. All had received professional alcohol/drug dependency treatment. All had received inpatient care, most for 3 months or more. All considered themselves 12-step group members and reported that they read 12-step literature and applied the steps to their lives on a daily basis. Participants provided an average of 25.56 days’ worth of data each (ranging from 10 to 33 days), with the average participant missing 0.8 days (ranging from 0 to 2 days). Notice that we used slightly different criteria than Zheng et al. (2013) to include the patients, leading to a slightly different sample in the current study.

*Tobacco use*. Daily tobacco use was measured with one item asking, “How many cigarettes did you smoke today?” Responses ranged from 0 (no cigarettes), 1 (1 or 2), 2 (2 to 5), 3 (5 to 10), 4 (half pack) to 5 (full pack plus).

*Substance use craving*. Daily substance use craving was measured with seven items modified from the Desires for Alcohol Scale and the Alcohol Urges Questionnaire to accommodate daily assessment and polydrug use. A sample item reads, “For a moment today I missed the feeling of drinking or drugging.” Responses were 1 (strongly disagree) to 5 (strongly agree).

*Negative affect*. Zheng et al. (2013) used the 10-item negative affect scale from the Positive and Negative Affect Schedule to assess daily negative affect. Example emotions including “Stressed,” “Upset,” “Scared,” “Hostile,” and “Irritable”. Responses ranged from 1, very slightly or not at all to 5, very much. Zheng et al. (2013) provided descriptive statistics for the three variables and the number of observations of each participant.

Our pre-processing step includes missing data imputation using the mean of the non-missing consecutive days, log-transformation of all three variables to improve normality, standardization to mean 0 and variance 1 and removal of linear trend for each time series for each patient by regressing individual variables against the “day” variable (i.e., from 1 to the total number of days for a participant) and taking the residuals.

### 3.2 Hyperpriors used in the model

We used weakly informative priors that are conjugate but almost flat on the parameter space [29]. In particular, we assume $\lambda_{1,k}^{2}$ and λ_2,*k*_, *k* = 1, …, *R*^2^ *p* follow Γ(1, 0.001) and Γ(1, 0.01) distributions respectively. The diagonal elements of Θ_*v*_ are assumed to follow Γ(1, 0.01). The precision matrix of the error term Λ is assumed to follow Wishart((*R* − 1)*I*_*R*_, 1). We assume the multiplicative variables (see S1 Appendix) follow a diffuse normal distribution: $\text{MVN}(0,100I_{R^{2}p})$.

### 3.3 Criterion to assess model performance

We use prediction accuracy as a criterion to assess the performance of the proposed sparse hierarchical VAR model. We compare it with the patient-specific VAR model used in Zheng et al. (2013) and regularized linear regression model, a state-of-the-art machine learning-based approach for patient level prediction modeling (see, for example, [35], [36]). In particular, all but the last daily record of each individual patient are used to train the model and the last daily record is used as a test set.

We use the posterior predictive distributions to predict the test data for the Bayesian model (see [26] for details about this procedure). Specifically, each individual sample of the 1-step ahead forecast for subject *n* = 1, …, *N* is generated by the posterior samples of the VAR coefficients and the error precision matrix:

$$\begin{array}{c} {\hat{y}}_{n,T_{n}+1}=\sum_{i=1}^{p}{\hat{A}}_{ni}y_{n,T_{n}}+{\hat{\epsilon }}_{n,T_{n}+1}, \end{array}$$ (19)

where ${\hat{\epsilon }}_{n,T_{n+1}}$ is drawn from a multivariate normal distribution with mean 0 and precision matrix equal to one posterior sample of Λ

$$\begin{array}{c} {\hat{\epsilon }}_{n,T_{n}+1}∼MN\left(0,\hat{\Lambda }\right). \end{array}$$ (20)

Posterior means and the 2.5-th and 97.5-th posterior percentiles of these posterior predictive samples are used as point and interval estimates of the prediction. For the patient-specific VAR model, maximum-likelihood estimates (MLE) are used to predict the test observation for each individual patient. For the regularized linear regression model, elastic-net regularization (with equal weights on *L*_1_ and *L*_2_ norms) was fitted for each patient to predict the current value of each individual variable based on the previous values of all three variables. 3-fold cross validation in conjunction with the “one standard error” criterion was used to train the model, select the tuning parameters and perform the future data prediction (the same procedure was utilized in [35]). Mean squared error (MSE) and coverage probability (obtained by the percentage of the test data points that are contained in their 95% predictive intervals) are used to assess prediction accuracy on the test set for all three models. Predictive intervals are obtained by the posterior quantiles for the the Bayesian model and by the bootstrap quantiles for the regularized linear regression model [37]. Note that using posterior modes for parameter estimation enforces sparsity and interpretation and using posterior means minimizes mean squared error (MSE) for the forecasting [26].

### 3.4 Results

#### 3.4.1 Posterior sampling and order selection

We fit two hierarchical VAR models with order one and two, respectively. The posterior sampling procedure was implemented in R. Specifically, we ran 10,000 iterations across 4 chains and used the first half of the iterations as warmup iterations. The second half was thinned by every 20 iterations to diminish the autocorrelation in the posterior samples, ending up with 500 samples per chain for posterior inference. All 4 chains mixed well and converged to similar stationary distributions for all parameters (the maximum of the Gelman-Rubin statistics [31] over all parameters in the model is 1.028). No serial correlations are found in the chains. The number of parameters for this example is about 300 for VAR(1) and 600 for VAR(2). (See S1 Fig for diagnostic plots of the mixing and S2 Fig for the autocorrelation of the chains for some of the parameters in the population-level coefficients).

For this particular example, the model with order one resulted in a smaller AIC (3027.951) and is favored. Since the second order model underperformed the first order model (AIC = 3466.319), we did not pursue higher orders. We note that Zheng et al. (2013) fit VAR(1) models to each individual patient. In fact, since there is one patient who has only 9 days of daily records in the training data, maximum likelihood estimation of models of higher order than one results in singularity problems requiring a Bayesian and/or regularized approach.

#### 3.4.2 Population-level coefficients and between-patient heterogeneity

**Fig 1:**
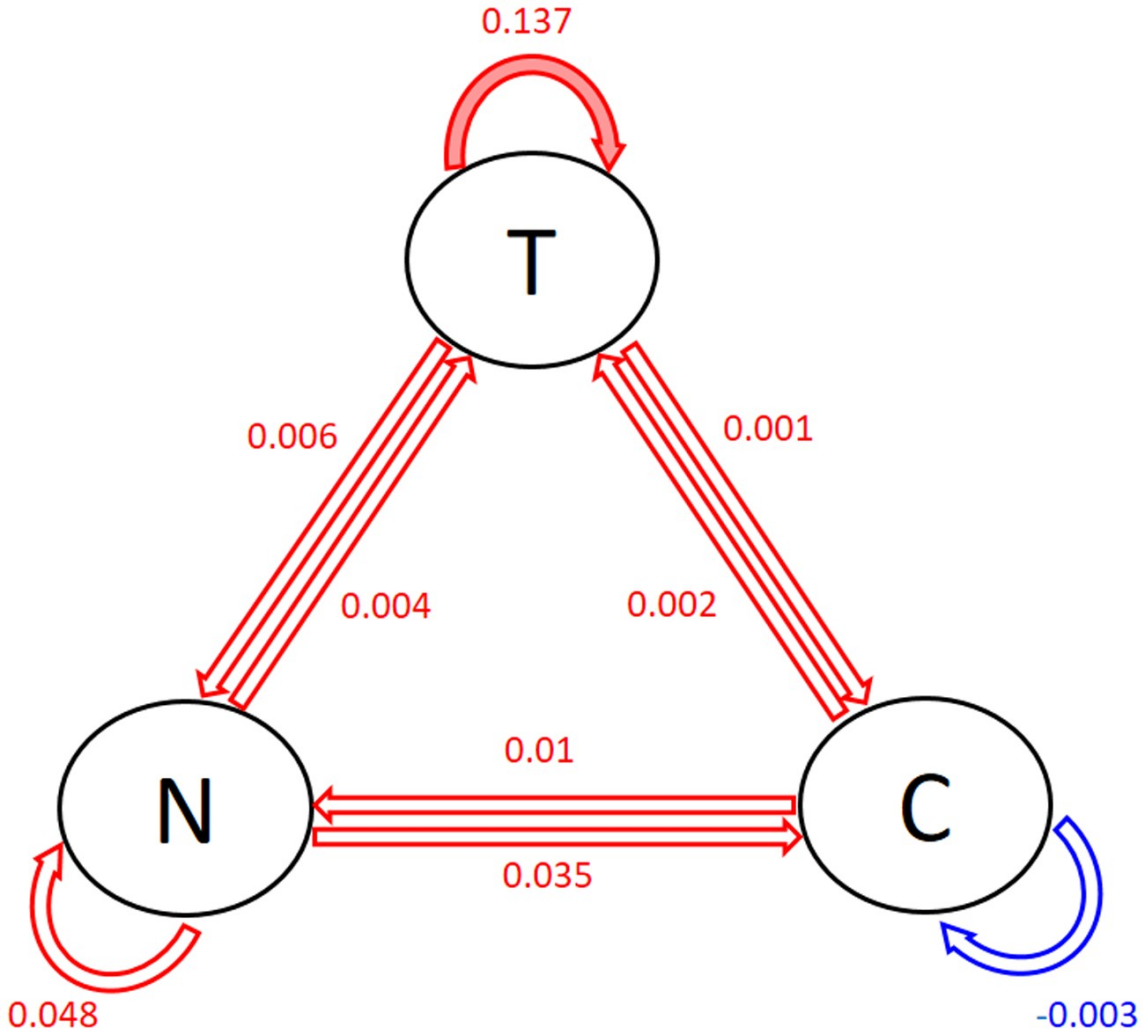
Posterior modes of the population-level coefficients. T = tobacco use, N = negative affect, C = craving. Red = positive, blue = negative. Solid = significant coefficients (red: *α* = 0.05; pink: *α* = 0.1), transparent = non-significant coefficients.

Fig 1 shows the posterior modes of the population-level coefficients, i.e., the posterior modes of the lag-1 associations between tobacco use, substance craving and negative affects. In the circles, T represents tobacco use, N represents negative affect and C represents craving. The arrows and the numbers next to the arrows display the values of the posterior modes of the lag-1 associations between the three variables. In particular, the red arrows show positive associations while blue arrow shows the negative one. The solid arrows represent the “significant” associations. Specifically, the pink arrow indicate that a 90% interval does not contain 0. The transparent arrows show the associations that are not significant. One can see from Fig 1 that tobacco use in the previous day has the strongest positive effect on the tobacco use in the current day. This suggests that when there is 1 unit increase in tobacco use in the previous day, there seems to be an increase (0.137 on the log scale) in tobacco use in the current day. The second and third strongest effects are negative affect in the previous day on the negative affect (0.048) and substance use craving (0.035) in the current day. Moreover, craving in the previous day has a small negative effect on itself in the current day (-0.003). These posterior modes of the lag-1 associations constitute a predictive model for predicting tobacco use, negative affect and craving in the current day based on the values of these variables in the previous day on the general population level.

#### 3.4.3 Patient-level coefficients

**Fig 2:**
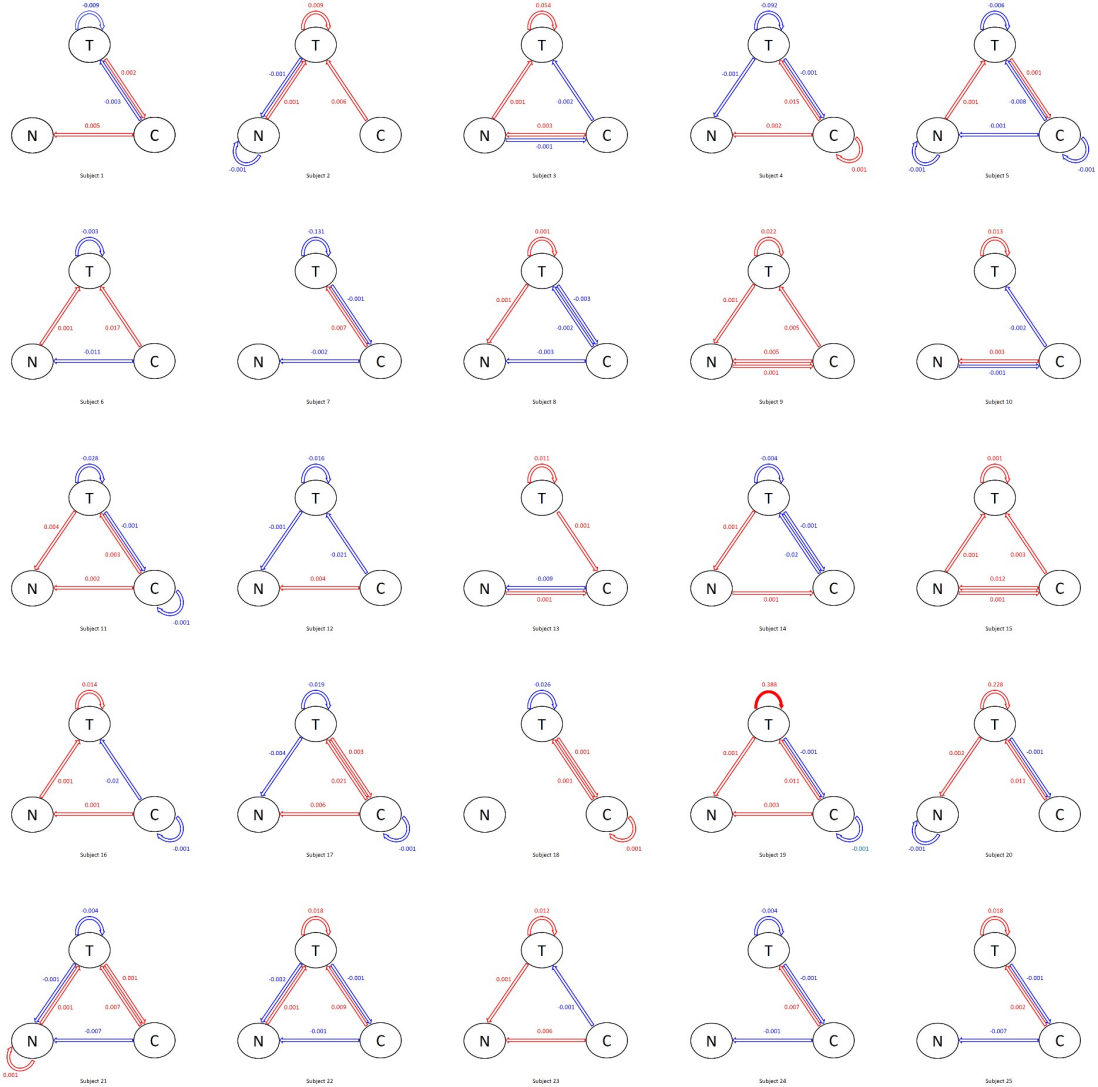
Posterior modes of the patient-level coefficients. T = tobacco use, N = negative affect, C = craving. Red = positive, blue = negative. Solid = significant coefficients (red: *α* = 0.05; pink: *α* = 0.1), transparent = non-significant coefficients.

Fig 2 shows the posterior modes of the patient-level coefficients. In these plots, the solid red arrow indicates that a 95% posterior interval does not contain 0. We see that several lag-1 associations between the three variables of interest are zero for most patients, including negative affect and craving in the previous day on themselves in the current day, as well as negative affect in previous days on tobacco use and craving in the current day. For the non zero associations, tobacco use in the previous day has an effect on tobacco use in the current day for all patients. Craving in the previous day also has an effect on tobacco use in the current day for almost all patients. These associations vary across different patients in terms of both sign and magnitude.

The advantage of the sparsity induced by the elastic-net prior over the existing patient-specific VAR model is that it reduces spurious relationships between the variables [38] and only selects the variables that are the most predictive for the outcome. Such selection of important variables vary across individual patients (for example, different patterns of associations between the variables can be found for patient 1, 2 and 18). This aids interpretation and provides the most important relationships between the three variables for each individual patient, leading to a useful insight for personalized treatment. In contrast, the patient-specific VAR model does not provide sparse estimates and thus no insights of important variable associations for individual patients are obtained. Further, our elastic-net prior utilizes adaptive shrinkage in the coefficient estimates (i.e., different *L*_1_ and *L*_2_ tuning parameters for different coefficients) while the regularized linear regression model uses the same tuning parameters for all coefficients. The latter leads to aggressive shrinkage in the coefficient estimates. In fact, most of the estimated coefficients given by the regularized linear regression model are zero, meaning that no variables are selected for the majority of the patients. Therefore, compared to the patient-specific VAR model that has no shrinkage and the regularized linear regression model that has aggressive shrinkage, our model provides decent amount of shrinkage and improves interpretation (see S1 Table for the proposed Bayesian VAR model, S2 Table for the patient-specific VAR model and S3 Table for the regularized linear regression model, respectively).

**Fig 3:**
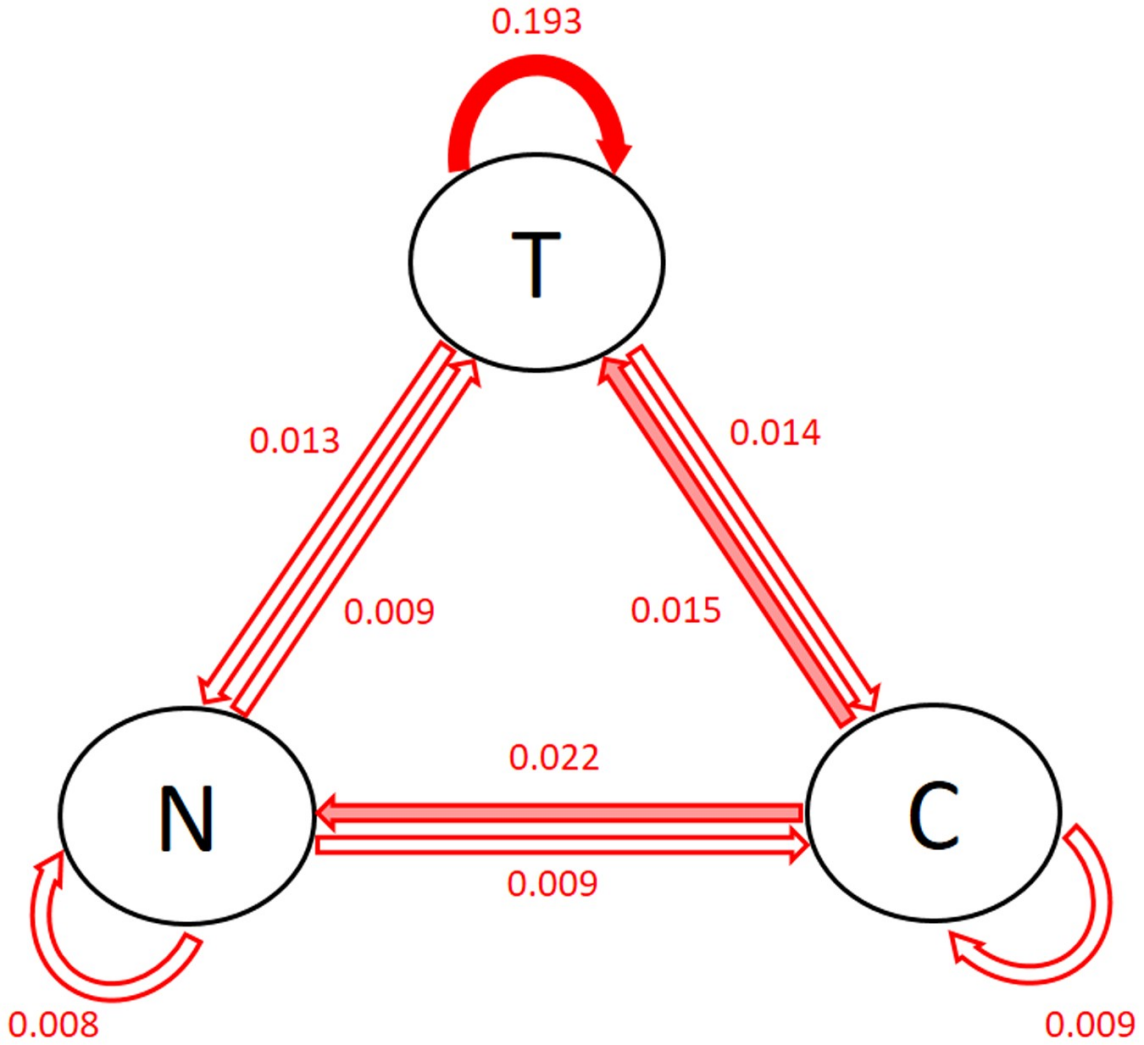
Posterior modes of the standard deviation of the patient-level coefficients. T = tobacco use, N = negative affect, C = craving. The first three largest standard deviations are shown in solid arrows.

Fig 3 shows the posterior modes of the standard deviation of the patient-level coefficients (i.e., the square root of the inverse of the diagonal elements of Θ_*v*_). The solid arrows show the associations with large patient-level heterogeneity: the largest one is the effect of previous tobacco use on current tobacco use, followed by previous craving on current negative affect and tobacco use. This is consistent with the findings in Fig 2. Note that the patient-specific VAR model and the regularized linear regression model do not provide quantification or interpretation of the patient-level heterogeneity.

#### 3.4.4 Gender and age group analysis

**Fig 4:**
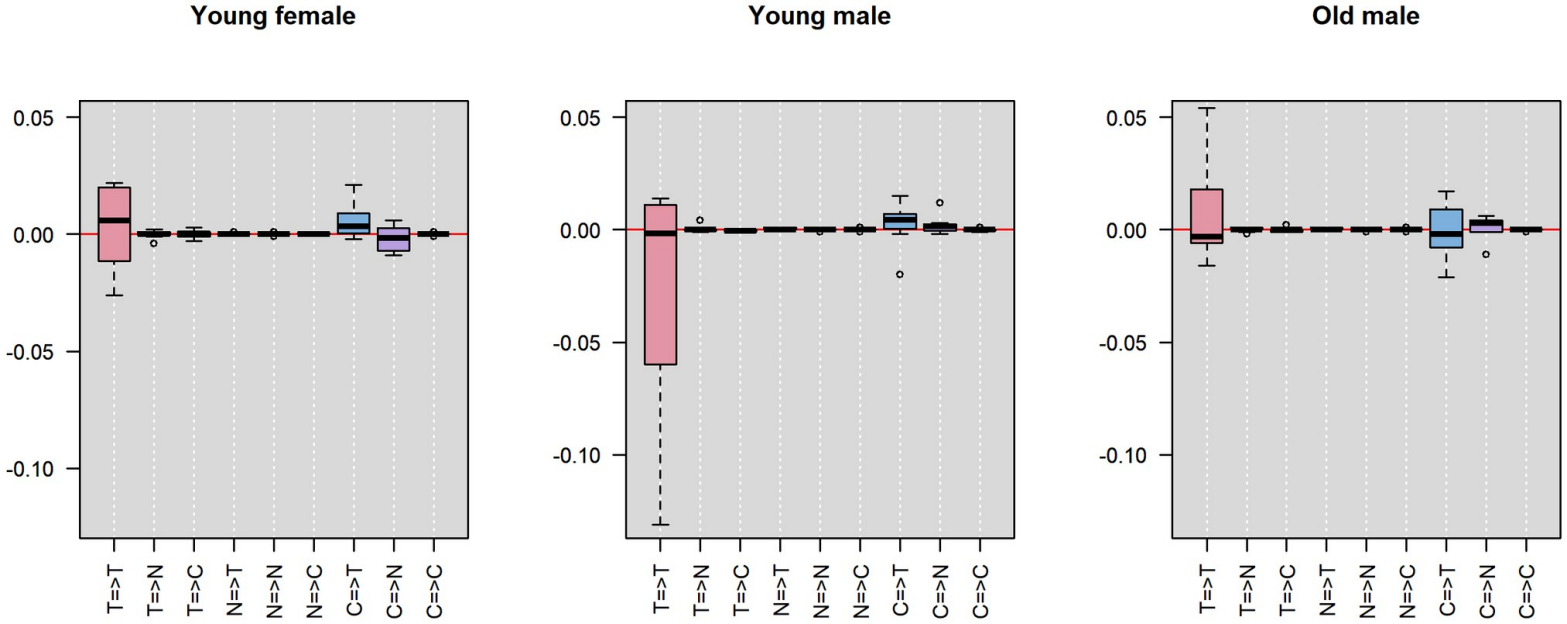
Distribution of the posterior modes of patient-level coefficients in different gender and age groups. T = tobacco use, N = negative affect, C = craving. Each panel represents one group. Each boxplot shows the lag-1 association between a pair of variables (the tick name “X=>Y” represents the lag-1 association of variable X in the previous day on variable Y in the current day).

To further investigate what constitutes the patient-level heterogeneity for each of the associations between the three variables, we separate the entire sample of 25 participants by gender and age into 3 groups: young female, young male and old male. We then look at the distribution of the posterior modes of the patient-level lag-1 coefficients (i.e., the arrows in the panels in Fig 2) within each group. The first two groups have age ranging from 18 to 21 years old and the third group from 22 to 32 years old (there is no female students who are older than 22 years old). This separation provides balanced sample sizes in the three groups: 8, 8 and 9, respectively. Fig 4 shows the distribution of the posterior modes of the patient-level lag-1 coefficients separated by the three groups. Each panel represents one group. Each boxplot represents the distribution of a distinct lag-1 association between a pair of variables (i.e., the tick name “X=>Y” represents the lag-1 association of variable X in the previous day on variable Y in the current day). Note that the y-axis range is truncated (removing one outlier from the distribution of the autocorrelation of tobacco use in the young female group and one in the old male group) for better visualization.

We can see that for the autocorrelation of tobacco use in the previous day on tobacco use in the current day (i.e., the pink box in the first column), the three groups tend to have similar medians with the young female group having a slightly higher median than the other two groups. However, the young male group has the greatest variance than the other two groups. Many of them also have strong negative patient-level coefficients, as apposed to the old male group, which has strong positive patient-level coefficients. This suggests that while increase in tobacco use in the previous day tends to increase tobacco use in the current day for the general population (as is displayed by Fig 1), the young male group tends to have less increase in the current day than the general population while the old male group tends to have greater increase in the current day than the general population. In other words, the continuous use of tobacco across days seems to be greater among older males.

Further, for the effect of craving in the previous day on negative affect in the current day (i.e., the purple box in the eighth column), the old male group seems to have a higher value than the other two groups. This suggests that for males older than 22 years, more craving in the previous day correlates to more negative affect in the current day than young females and young males. Moreover, for the effect of craving in the previous day on tobacco use in the current day (i.e., the blue box in the seventh column), the old male group has lightly greater variance than the other two groups. This suggests that the effect of craving in the previous day on tobacco use in the current day can be more different between individual old males than between the younger participants.

#### 3.4.5 Prediction accuracy

Table 1 shows the MSE’s of the proposed Bayesian hierarchical model compared with the patient-specific VAR model and the elastic-net regularized linear regression model (the % reduction in MSE is displayed in italic font). We see that the hierarchical model improved the accuracy of the prediction on both competitor models. The overall mean square error averaged over all three variables is reduced, respectively, by 25% and 8% using the Bayesian model relative to the patient-specific VAR model and the regularized linear regression model. For the individual variables, predictive accuracy is the same or better for all three variables with the mean square errors reduced by 17% and 12% for tobacco use, 4% and 0% for negative affect, and 38% and 12% for craving. This shows the advantage in terms of predictive accuracy of the hierarchical model which pools the information across all patients and induces regularization in parameter estimation.

**Table 1:** **Application 1:** MSE of the Bayesian hierarchical VAR model, the patient-specific VAR model and the regularized linear regression model.

Model	Overall MSE	MSE by Variable		
		T	N	C
Bayesian VAR model	.722	.459	.704	1.00
Patient-specific VAR model	.964	.554	.731	1.608
*% Reduction*	*25.1%*	*17.1%*	*3.7%*	*37.7%*
Regularized linear model	.787	.524	.705	1.133
*% Reduction*	*8.3%*	*12.3%*	*0.1%*	*11.5%*

T = tobacco use, N = negative affect, C = craving.

**Fig 5:**
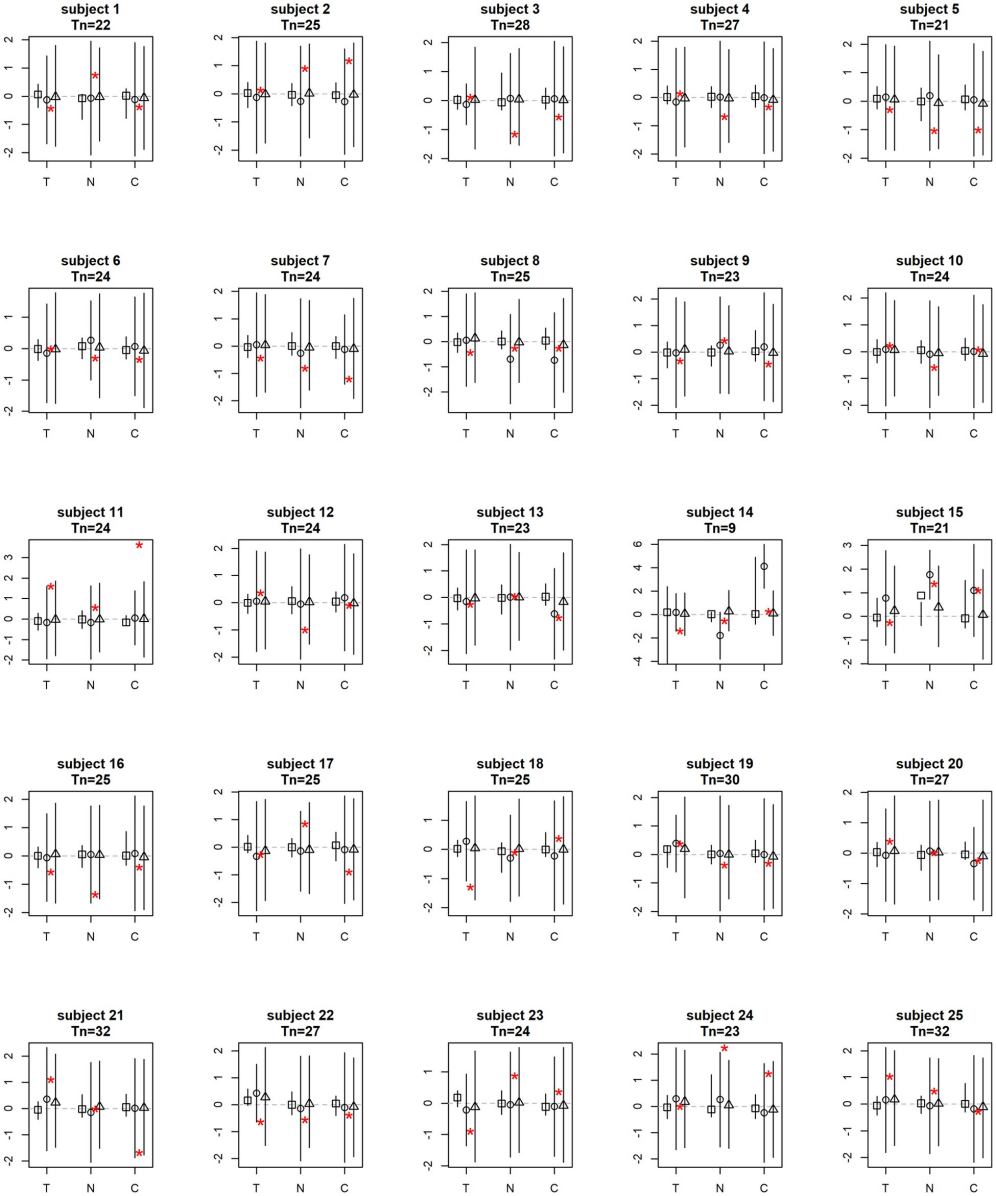
Posterior means and 95% intervals for the 1-step ahead prediction. T = tobacco use, N = negative affect, C = craving. The squares represent the regularized linear regression model, the circles represent MLE’s and the triangles represent the Bayesian estimates. The lines show the 95% intervals. Red stars are the true future observations. Number of observations (*T*_*n*_) for each subject is shown in the subtitle.

Fig 5 shows the prediction intervals for all subjects using the proposed Bayesian model, the patient-specific maximum-likelihood estimates and the regularized linear regression model. The squares represent the regularized linear regression model, the circles represent the patient-specific MLE’s, and the triangles represent the Bayesian estimates. The lines going through the dots show the 95% intervals. Red stars show the true test observations. The numbers of time points in the training data for each patient are shown as well. We see that for moderately large sample sizes (above 20 time points), the point estimates are quite similar for all three models. However, for the subject with small sample size (subject 14 has only 9 training observations), Bayesian prediction is remarkably better than the MLE for craving. Both the Bayesian model and the regularized linear regression model enforce elastic-net regularization so the point estimates obtained by both models are shrink towards 0.

The estimated coverage probabilities of 95% predictive intervals are, respectively, 97%, 93% and 33.3% for the Bayesian model, the patient-specific VAR model and the regularized linear regression model. For a nominal level of 95%, we would expect about 95% of the predictive intervals to contain the true values. Both the Bayesian model and the patient-specific VAR model are close to the nominal level, whereas the regularized linear regression model is substantially undercovered. Further, 68% of the Bayesian intervals are narrower than those given by the patient-specific VAR model. This shows that the Bayesian method can not only provide more accurate point estimates but also a more satisfactory range estimate as well. Note that the majority of the intervals (more than 95%) given by regularized linear regression model are narrower than the other two methods. Both the undercover issue and the short length of these intervals are due to the aggressive nature of the shrinkage of this model.

## 4 Application 2: Predicting functional somatic symptoms (FSS) and psychological discomforts

Approximately 20% to 50% of the physical symptoms presented in primary care and hospital settings cannot be fully explained by organic pathology [39] [40] [41]. These symptoms are commonly referred to as functional somatic symptoms (FSS). Previous studies in epidemiology have suggested greater stress [42] [43] [44] and other psychological discomforts [45] [46] in people who suffer from FSS’s than those who do not. Utilizing patient diary databases, a few studies have shown cross-relationship between stress and FSS’s in both directions within subjects over time [47] [48].

To investigate bidirectional relationship and temporal precedence between stress and FSS’s within individual patients, van Gils et al. (2014) adopted a VAR modeling framework on data from a diary study with patients who had multiple, persistent FSS’s, including external and internal body pain and other symptoms related to the autonomic nervous system [23]. A patient-by-patient Granger causality analysis of stress and several FSS’s showed that an increase in one or more FSS’s were found to be significantly predictable by preceding (mostly first-lag) increase in stress for 30% of the patients, and reverse association in 15% of the patients. Substantial between-patient heterogeneity in the lagged associations between the FSS’s and stress was also reported. We re-analyze the same dataset using the proposed model.

### 4.1 Data and the preprocessing

26 patients with persistent FSS’s and psychological discomforts were recruited by medical practitioners in a 12-week study between January 2004 and February 2006. Standard handheld personal digital assistant (PDA) computers with the Palm operating system were used to record daily diary on 14 FSS’s (muscle pain, joint pain, back pain, headache, abdominal pain, pelvic pain, bowel symptoms, dyspepsia, nausea, tight throat, chest pain, weakness, numbness and palpitations) and 5 psychological discomforts (stress, fatigue, anxiety, depression and illness concern). Patients entered 1-3 records on each day, with each data entry consisting of 3 most severe symptoms out of the 14 FSS’s and all 5 psychological discomfort measures (all patients entered the same 3 symptoms during the entire study period). All participants were fully anonymized before accessing the data.

*FSS’s*. Diary questions with respect to FSS’s were phrased as follows: “How much have you been bothered by symptom X? Please mark a point on the line between severe symptom X and no symptom X at all.” (1-150).

*Psychological discomforts*. The level of stress and other psychological discomforts were assessed using the following question: “How stressful (fatigue, anxiety-arousing, depressing, illness-concerning) are people and things around you? Please mark a point on the line between very stressful (fatigue, anxious, depressed, illness-concerned) and not stressful (fatigue, anxious, depressed, illness-concerned) at all.” (1-150).

Since only 3 out of the 14 FSS’s were recorded for each patient, most of the FSS’s were recorded by a very small number of patients (10 out of the FSS’s were recorded by less than 3 patients and 1 FSS by 9 patients). We therefore use subsets of the data on 4 of the FSS’s (headache, joint pain, bowel symptoms, muscle pain) that were recorded by the greatest number of patients (*N* = 12, 12, 10, 15, respectively). All patients in these subsets are included in the analysis. The age of the patients included in the four models ranges from 29 to 59 years old and there is only 1 male in the first 3 models and 4 males in the fourth model. For each of the 4 FSS’s, we extend the previous work where the relationship between one FSS and stress was considered [23], and fit the proposed Bayesian VAR model on the FSS and *all* 5 psychological discomfort variables. This results in 4 models, each of which considers 6 variables. The average (range) length of diary records for the 4 FSS’s are, respectively, 90 (64-126), 89 (64-131), 86 (64-109), 90 (83-126) days, with average (range) number of missing daily records 9 (0-37), 9.5 (0-47), 6 (0-24), 4.4 (0-37), respectively.

The pre-processing of the data included: 1) averaging of the variables for each day and each patient to ensure one observation per day per patient, if there are more than one records entered on the same day for the same patient; 2) missing value imputation using moving average imputation embedded in R package ‘imputeTS’ (https://cran.r-project.org/web/packages/imputeTS/index.html); 3) log-transformation of each of the 6 variables to improve normality; 4) standardization to mean 0 and variance 1 and removal of linear trend for each time series for each patient (the same as in Section 3). Note that we use a slightly more elaborate imputation procedure here because there are more missing observations than those in the first application.

We use the same posterior sampling strategy and hyperpriors in Section 3 for this application. We use the last 10 observations of each patient as test data and the remainder training data. 1-step ahead forecasting was performed using the same technique in Section 3 and *h*-step ahead forecasting, *h* = 2, …, 10, was performed using the recursive algorithm (see [26] for details). Posterior modes of the parameters are used for statistical inference. Assessment of prediction accuracy (i.e., MSE of the posterior means and coverage probabilities of 95% predictive intervals) and model order selection (i.e., AIC) are the same as Section 3.

### 4.2 Results

#### 4.2.1 Posterior sampling and order selection

For each of the 4 models, we ran 20,000 iterations across 4 chains and used the second half of the iterations (thinned by every 40 iterations, yielding 250 samples per chain) for posterior inference. All chains mixed well and converged to the stationary distribution (the maximums of the Gelman-Rubin statistics over all parameters are, respectively, 1.016, 1.023, 1.018, 1.026 for the 4 FSS’s). No serial correlations are found in the chains. VAR(1) models outperformed VAR(2) models with smaller values of AIC across all 4 models (the VAR(1) models for the 4 FSS’s have AIC values 5963.7, 6166.4, 4725.1, 7419.5, respectively, and the VAR(2) models 7054.0, 7212.4, 5719.7, 8670.4). We therefore favor VAR(1) models in this application (see S3 to S10 Figs for diagnostic plots for the first 9 parameters in the population-level coefficients for each of the 4 models).

#### 4.2.2 Population-level coefficients

**Fig 6:**
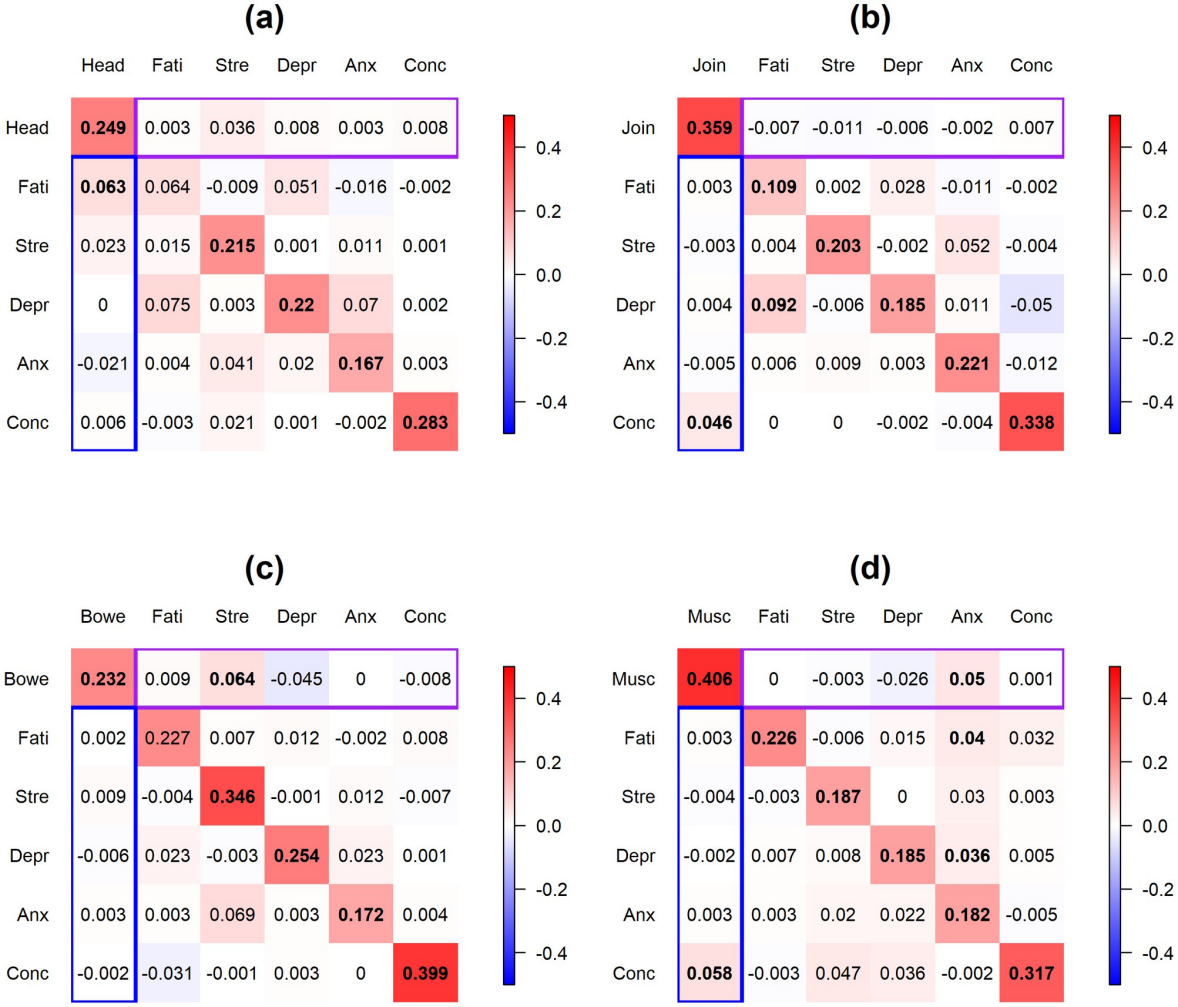
**Application 2:** Posterior modes of the population-level coefficients for the 4 FSS’s. Panel (a)-(d) display FSS = headache, joint pain, bowel symptoms and muscle pain, respectively. The coefficient at the *r*-th row and the *c*-th column in each panel represents the effect of the *c*-th variable in the previous day on the *r*-th variable in the current day. Significant coefficients (at level *α* = 0.1) are in bold font. Purple box on each panel represents the effect of the psychological discomforts in the previous day on the FSS in the current day; blue box represents the effect of FSS in the previous day on the psychological discomforts in the current day. Head = headache, Join = joint pain, Bowe = bowel symptoms, Musc = muscle pain, Fati = fatigue, Stre = stress, Depr = depression, Anx = anxiety, Conc = illness-concern. Color scale ranges from -0.5 to 0.5.

Fig 6 displays the posterior modes of the population-level coefficients for the 4 models (panel (a)-(d) represent FSS = headache, joint pain, bowel symptom and muscle pain, respectively). The coefficient at the *r*-th row and the *c*-th column in each panel represents the effect of the *c*-th variable in the previous day on the *r*-th variable in the current day. Significant coefficients (at level *α* = 0.1) are in bold font. Purple box on each panel represents the effect of the psychological discomforts in the previous day on the FSS in the current day; blue box represents the reverse effect of FSS in the previous day on the psychological discomforts in the current day.

Inspection of Fig 6 indicates that most FSS’s and psychological discomfort variables have positive effects on themselves. Most of the cross-relationships between FSS’s and psychological discomforts are positive and the magnitudes are smaller than the autocorrelations of the variables. The strongest cross-associations between FSS’s and psychological discomforts include the effect of headache in the previous day on fatigue in the current day for patients who reported headache, the effect of joint pain in the previous day on illness concern in the current day for patients who reported joint pain, the effect of stress in the previous day on bowel symptoms in the current day for patients who reported bowel symptoms, and the effect of anxiety/muscle pain in the previous day on muscle pain/illness concern in the current day for patients who reported muscle pain. Moreover, there are significant associations between the psychological discomfort variables, including the effect of fatigue on depression for patients who reported joint pain and anxiety on fatigue and depression for patients who reported muscle pain.

#### 4.2.3 Between-patient heterogeneity

**Fig 7:**
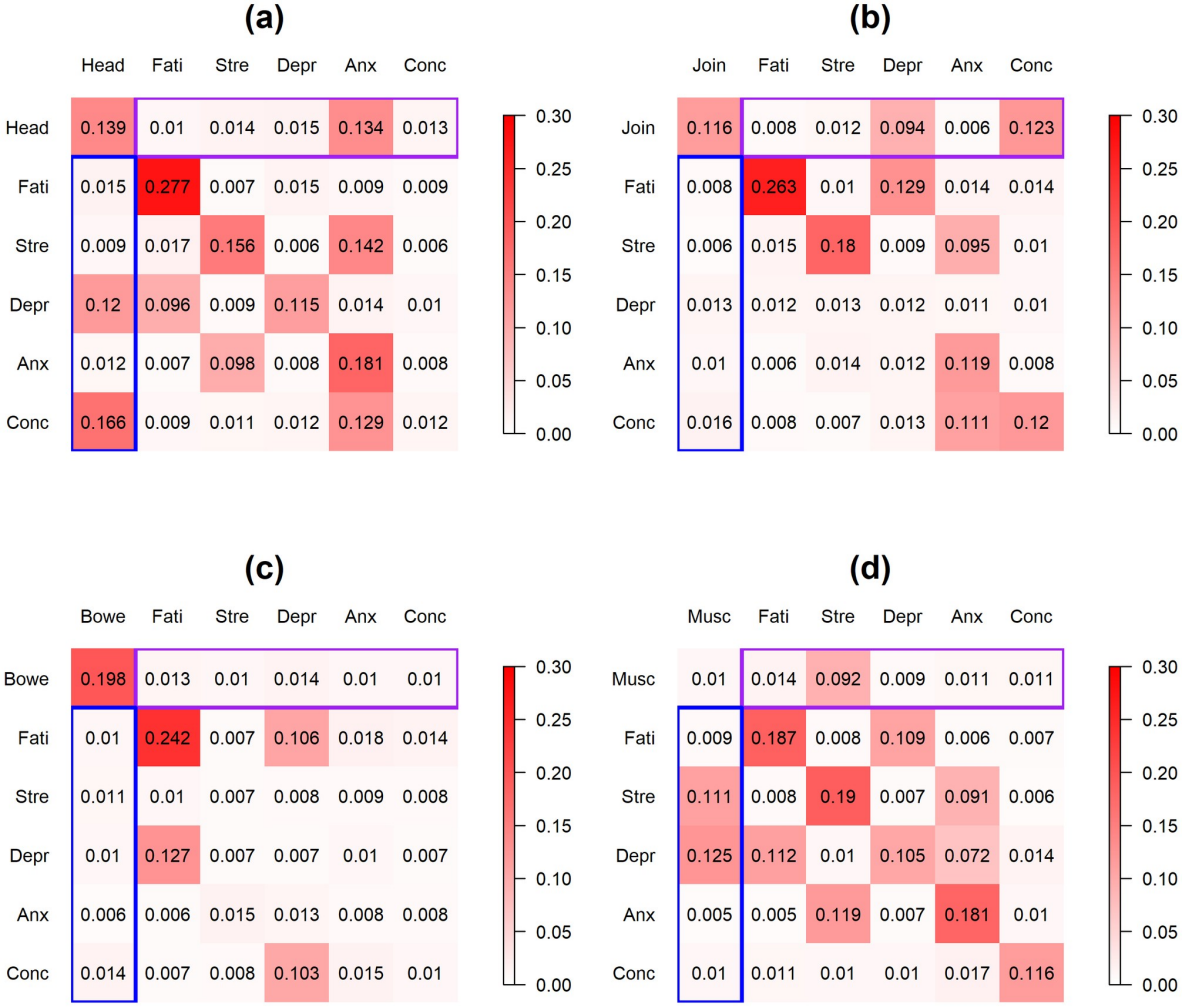
**Application 2:** Posterior modes of the standard deviations of the patient-level deviations. Posterior modes of the standard deviations of the patient-level deviations for the 4 FSS’s. Panel (a)-(d) display FSS = headache, joint pain, bowel symptoms and muscle pain, respectively. The number at the *r*-th row and the *c*-th column in each panel represents the standard deviation of the effect of the *c*-th variable in the previous day on the *r*-th variable in the current day. Purple box on each panel represents between-patient heterogeneity in the effect of the psychological discomforts in the previous day on the FSS in the current day; blue box represents the between-patient heterogeneity in the effect of FSS in the previous day on the psychological discomforts in the current day. Head = headache, Join = joint pain, Bowe = bowel symptoms, Musc = muscle pain, Fati = fatigue, Stre = stress, Depr = depression, Anx = anxiety, Conc = illness-concern. Color scale ranges from 0 to 0.3.

Fig 7 displays the posterior modes of the standard deviations of the patient-level deviations for the 4 models (Panel (a)-(d) represent FSS = headache, joint pain, bowel symptom and muscle pain, respectively). The highest between-patient heterogeneity lies in the autocorrelation of fatigue for patients who reported headache, joint pain or bowel symptoms. For cross-relationships between the FSS’s and the psychological discomforts, the highest between-patient heterogeneity was present in the effect of headache on illness-concern and depression and the effect of anxiety on headache for patients who reported headache, the effect of illness-concern and depression on joint pain for patients who reported joint pain, and the effect of muscle pain on stress and depression and the effect of stress on muscle pain for patients who reported muscle pain. The last finding is consistent with the previous studies which have found substantial between-subject heterogeneity in the association between stress and FSS’s [49]. Note that neither the patient-specific VAR model nor the regularized linear regression model quantifies the between-patient heterogeneity.

#### 4.2.4 Age group analysis

**Fig 8:**
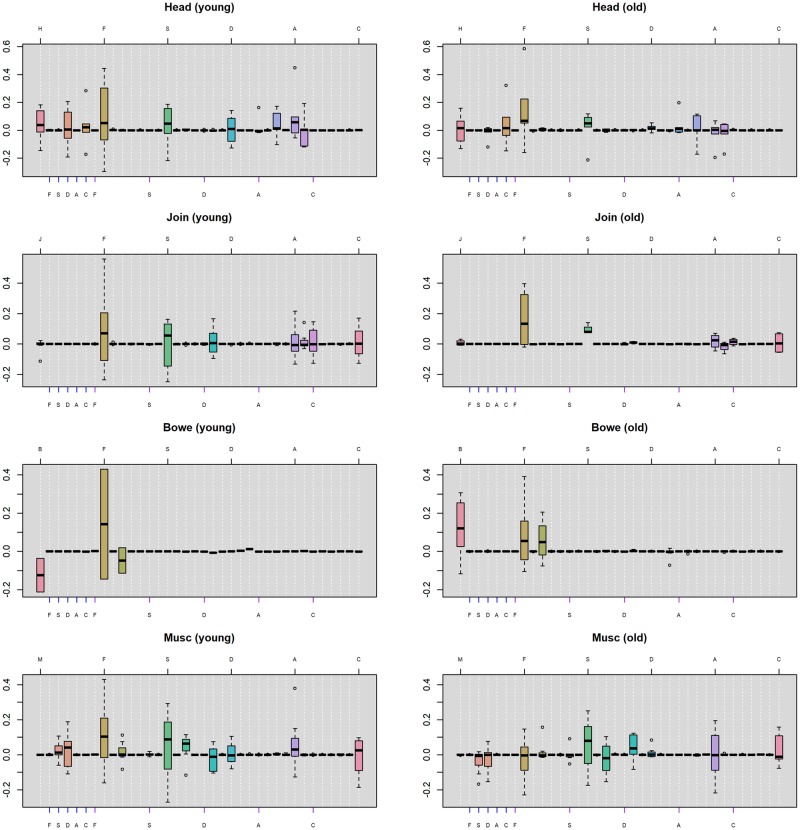
**Application 2:** Distribution of posterior modes of the patient-level coefficients. Each boxplot displays the patient-level coefficients of the lag-1 associations between the FSS’s and psychological discomforts. The left and right columns represent the young group (less than or equal to 44 years old) and the old group (greater than 44 years old). The ticks on the top line in each panel mark the lag-1 autocorrelations of the FSS and the psychological discomforts. The blue ticks on the bottom line mark the effect of the FSS in the previous day on psychological discomforts in the current day, and the purple ticks mark the reverse effect of psychological discomforts on FSS. H = headache, J = joint pain, B = bowel symptoms, M = muscle pain, F = fatigue, S = stress, D = depression, A = anxiety, C = illness-concern.

To further investigate what constitutes the substantial between-patient heterogeneity, we separate the patients into two groups by age: a young group with age ranging from 29 to 44 years old and an old group with age ranging from 44 to 59. The sample sizes for the younger patients are 6, 8, 2 and 7 for the 4 FSS’s respectively (note that we choose 44 years old to be the cutoff in order to ensure most FSS’s to have balance sample sizes of the two groups). We did not separate the patients by gender because the majority of the patients are female (there is only 1 male in the first 3 FSS models and only 4 males in the fourth FSS model) and separation by gender will lead to very imbalanced subsets. Fig 8 displays the distribution of the posterior modes of patient-level coefficients for the two groups. In each panel, the boxplots represent the lag-1 patient-level coefficients between the FSS and the psychological discomforts. The ticks on the top line mark the lag-1 autocorrelations of the FSS and the psychological discomforts. The blue ticks on the bottom line mark the effect of the FSS in the previous day on each individual psychological discomforts in the current day. The purple ticks mark the reverse effect of psychological discomforts on FSS (the colors of the ticks are consistent with Figs 6 and 7).

Inspection of Fig 8 indicates that the young group has a higher variability in the autocorrelations and the cross-associations of the variables in general. Specifically, younger patients have greater variability in the autocorrelation of fatigue (i.e., the brown box in the eighth column in each panel) than the old group across all 4 FSS’s. In other words, the large between-patient heterogeneity in the autocorrelation of fatigue (as is shown in Fig 7) is mainly explained by the variabilities in the young group. This suggests that while some young patients develop much greater fatigue in the current day after experiencing increased fatigue in the previous day, others may have much less fatigue in the current day. In contrast, the old group tends to have a smaller increment in fatigue in the current day after experiencing increased fatigue in the previous day for most of the FSS’s. Moreover, for the autocorrelation of bowel symptoms (i.e., the pink box in the first column in the panels in the third row), the young group has a majority of negative coefficients whereas the old group has a majority of positive coefficients. This suggests that when the bowel symptoms increased in the previous day, the young group tends to have less bowel symptoms in the current day than the general population, whereas the old group tends to have more bowel symptoms than the general population in the current day.

For the cross-relationships, the young group seems to have larger variability than the old group on the effect of previous-day headache on current-day depression (i.e., the light pink box in the fourth column in the panels in the first row), whereas the old group seems to have a larger variability than the young group on the effect of previous-day headache on current-day illness-concern (i.e., the orange box in the sixth column in the panels in the first row). Further, the young group seems to have a majority of positive effects of previous-day stress on current-day muscle pain while the old group has a majority of negative effects (i.e., the pink box in the third column in the panels in the fourth row). While these cross-associations have been shown to have large between-patient heterogeneity (as is indicated by Fig 7), different age groups tend to contribute different amount of variability in these cross-relationships.

#### 4.2.5 Prediction accuracy

**Fig 9:**
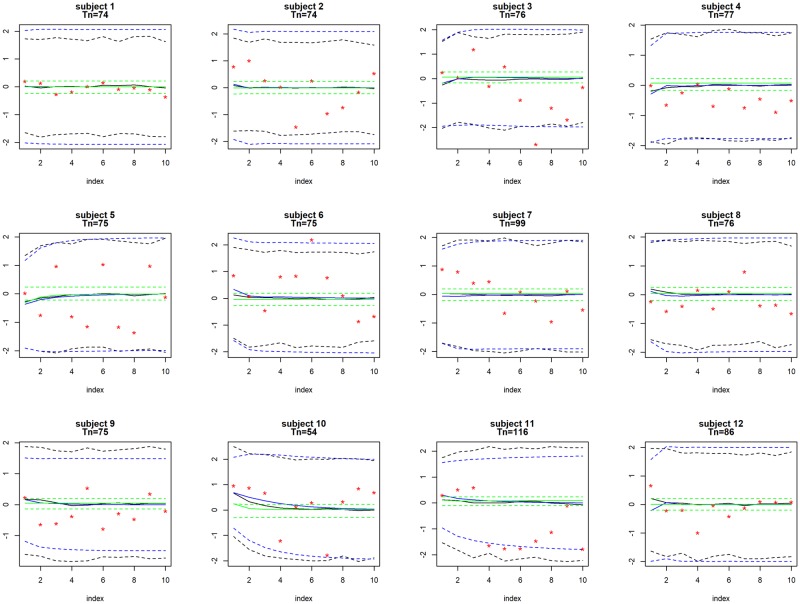
**Application 2:** Posterior means and 95% intervals for predicting headache. Posterior means and 95% intervals for predicting headache for all patients who reported this FSS using the Bayesian VAR model, the patient-specific VAR model and the regularized linear regression model. Each panel represents one patient (the index shows the *h*-step forecasting, *h* = 1, …, 10). The black, blue and green lines display, respectively, the prediction given by the Bayesian model, the patient-specific VAR model and the regularized linear regression model. Solid lines represent the point estimates and dashed line the predictive intervals given by the methods. Red dots show the true values of the test data points. Number of observations (*T*_*n*_) for each patient is displayed in each panel.

Table 2 displays the performance of predicting the10 test data points using the Bayesian VAR model, the patient-specific VAR model and the regularized linear regression model for the 4 FSS’s. Estimated MSE’s and coverage probabilities are shown for each single FSS. % reduction in MSE and % shorter predictive intervals given by the Bayesian model relative to the other two models are shown in italic font. Fig 9 displays the *h*-step forecasting, *h* = 1, …, 10, on headache for all patients who reported this FSS. The black, blue and green lines display, respectively, the prediction given by the Bayesian model, the patient-specific VAR model and the regularized linear regression model. Solid lines represent the point estimates and dashed lines the predictive intervals given by the three methods. Red dots show the true values of the test data points.

**Table 2:** **Application 2:** Prediction accuracy of the Bayesian model, the patient-specific VAR model and the regularized linear regression model.

	Headache	Joint Pain	Bowel Symptoms	Muscle Pain
	MSE			
Bayesian VAR model	0.741	0.695	0.568	0.698
Patient-specific VAR model	0.744	0.698	0.584	0.704
*% Reduction*	*0.38%*	*0.52%*	*2.64%*	*0.90%*
Regularized linear regression model	0.790	0.749	0.609	0.755
*% Reduction*	*6.16%*	*7.29%*	*6.68%*	*7.64%*
	Coverage probability			
Bayesian VAR model	0.963	0.964	0.97	0.964
Patient-specific VAR model	0.954	0.961	0.968	0.958
*% Shorter interval*	*64%*	*65%*	*61%*	*68%*
Regularized linear regression model	0.214	0.189	0.22	0.244
*% Shorter interval*	*0%*	*0%*	*0%*	*0%*

We can see that both the Bayesian model and the patient-specific VAR model performed similarly, with the Bayesian model slightly better across all 4 FSS’s. This is probably due to the fact that the number of observations (*T*_*n*_, *n* = 1, …, *N*) for each patient in the training data is much greater than that in Section 3, so the Bayesian model utilized much more information contained in the data than in the prior distributions, leading to similar behavior to the maximum-likelihood approach. Nevertheless, the Bayesian model still outperformed the patient-specific models in point estimates due to the pooling among patients. Further, the regularized linear regression model underperformed the other two methods. In fact, the cross-validation procedure always selected large values for the tuning parameters (i.e., strong regularization in the estimation), leading to the models that only contained intercepts. This resulted in less satisfactory prediction and low interpretability for most of the cases.

For the estimated coverage probability, both the Bayesian model and the patient-specific VAR model tend to slightly overcover (with the Bayesian model slightly more overcovered), but the Bayesian model provides shorter predictive intervals due to the shrinkage imposed by the priors. Similar to the first application, the regularized linear regression model substantially undercover across all 4 FSS’s.

## 5 Discussion

In summary, we proposed a novel Bayesian hierarchical VAR model for PLPM. Our model simultaneously estimates population-level and patient-level coefficients, as well as estimating between-patient heterogeneity. While previous methods either imposes no sparsity (e.g., the patient-specific VAR model) or too much sparsity (e.g., the regularized linear regression model), our model selects the most important predictors that lead to better interpretability and visualization as well as higher predictive accuracy. Further, while previous studies utilized ad-hoc approaches such as clustering analysis to qualitatively identify patterns in subgroups and no quantification of between-patient difference is provided, our model quantifies the between-patient heterogeneity without loss of information.

Our primary objective was to evaluate the hierarchical approach to patient diary modeling as compared with the widely-used patient specific models. We included an elastic-net regularized linear model to provide an alternative benchmark. We note that the diary application could utilize other approaches such as neural networks, support vector machines or random forest, although the elastic-net approach has been highly competitive in many applications (see, for example, [50], [51]).

In the examples, we used weakly informative hyperpriors which are conjugate but almost flat on the parameter space. This provides convenient derivation of the conditionals used in the Gibbs sampler while enforcing little prior information. We also tried different values of the hyperparameters but these yielded very similar results.

Furthermore, in the examples, we used posterior modes as parameter estimates for better interpretability and visualization. However, we also tried posterior median. Due to the fact that the posterior distributions are symmetric and unimodal for most of the parameters, the results were very similar in terms of both parameter estimation and future data prediction, although sparsity in the parameter estimation then no longer exists.

Moreover, we used a relatively simple missing data imputation scheme in the applications. This is to facilitate computational speed. Certainly more elaborate imputation procedure could be employed. Due to the fact that there are only a very small portion of missingness in both applications, different imputation approaches should not lead to drastically different results.

We also notice that the point estimates of the future observations in both applications are close to zero, while the true values can be quite far away from zero. We suspect two reasons for this phenomenon: 1) the Bayesian method has bias-variance trade-off, that is, it increases bias by shrinking the prediction towards zero but greatly reduces variance, leading to smaller prediction errors in general; 2) the test data contains a lot of variability and both existing state-of-the-art models and the proposed model need more covariates to improve prediction. In future work, we hope to include more covariates (including demographic variables, time information such as weekdays or weekends, and activity information in the previous day) that may be predictive about the outcomes in order to improve the prediction accuracy for cases like this.

In addition, due to the limited duration of observation in the first application, we used only 1 day as test data for each patient. As such, this probably has limited clinical utility. For the second application, however, 10 days (i.e., approximately 16% of the observation duration for patients with a smaller number of diary records) are used as test data. We believe this can prove meaningful in clinical practice.

Lastly and equally importantly, in the current application, we derive associations, rather than causal interpretations, between the variables. If causation is warranted, confounding must be addressed in the model. Adding covariates, for example, might allow one to address confounding and derive a more interpretable model for causal inference.

All code that generated the analyses in the paper and the data used in the first application are provided as online supplementary files (see S1 Rcode and S1 Data, respectively).

## Supporting information

S1 Appendix Parameter expansion. We implemented a Gibbs sampler algorithm to draw from the posterior distribution of the proposed model. We adopt a parameter expansion strategy to cope with high correlations among the parameters.
(PDF)

S2 Appendix Full conditional distributions. To draw from posterior distribution using Gibbs sampler, one draws each parameter from its conditional distribution given all other parameters at their current values. We provide full conditionals for all parameters.
(PDF)

S1 Fig Mixing of chains in the posterior sampling for population-level coefficients for Application 1. w1 to w9 indicate the 9 population-level coefficients. The 4 chains are displayed by different colors in each panel.
(TIF)

S2 Fig Autocorrelation of the chains in the posterior sampling for population-level coefficients for Application 1. w1 to w9 indicate the 9 population-level coefficients. The 4 chains are displayed by different colors in each panel.
(TIF)

S3 Fig Mixing of chains in the posterior sampling for population-level coefficients for Application 2 FSS headache. w1 to w9 indicate the first 9 population-level coefficients. The 4 chains are displayed by different colors in each panel.
(TIF)

S4 Fig Autocorrelation of the chains in the posterior sampling for population-level coefficients for Application 2 FSS headache. w1 to w9 indicate the 9 population-level coefficients. The 4 chains are displayed by different colors in each panel.
(TIF)

S5 Fig Mixing of chains in the posterior sampling for population-level coefficients for Application 2 FSS joint pain. w1 to w9 indicate the first 9 population-level coefficients. The 4 chains are displayed by different colors in each panel.
(TIF)

S6 Fig Autocorrelation of the chains in the posterior sampling for population-level coefficients for Application 2 FSS joint pain. w1 to w9 indicate the 9 population-level coefficients. The 4 chains are displayed by different colors in each panel.
(TIF)

S7 Fig Mixing of chains in the posterior sampling for population-level coefficients for Application 2 FSS bowel symptoms. w1 to w9 indicate the first 9 population-level coefficients. The 4 chains are displayed by different colors in each panel.
(TIF)

S8 Fig Autocorrelation of the chains in the posterior sampling for population-level coefficients for Application 2 FSS bowel symptoms. w1 to w9 indicate the 9 population-level coefficients. The 4 chains are displayed by different colors in each panel.
(TIF)

S9 Fig Mixing of chains in the posterior sampling for population-level coefficients for Application 2 FSS muscle pain. w1 to w9 indicate the first 9 population-level coefficients. The 4 chains are displayed by different colors in each panel.
(TIF)

S10 Fig Autocorrelation of the chains in the posterior sampling for population-level coefficients for Application 2 FSS muscle pain. w1 to w9 indicate the 9 population-level coefficients. The 4 chains are displayed by different colors in each panel.
(TIF)

S1 Table Patient-level coefficients obtained by the Bayesian VAR models. Each row is for one patient and each column is for one VAR coefficient.
(PDF)

S2 Table Patient-level coefficients obtained by the patient-specific VAR model. Each row is for one patient and each column is for one VAR coefficient.
(PDF)

S3 Table Patient-level coefficients obtained by the regularized linear regression model. Each row is for one patient and each column is for one VAR coefficient.
(PDF)

S1 Rcode R code to generate all analysis. This zip file contains all R code to generate the analysis, including the functions of the posterior sampling procedure for the proposed Bayesian model and the analysis on the real data.
(ZIP)

S1 Data Data for Application 1. This zip file contains the data used in the first application. All datasets are in csv format.
(ZIP)
