# Supplementary material for: Bayesian hierarchical vector autoregressive models for patient-level predictive modeling
Source: PLoS One. 2018 Dec 14;13(12):e0208082. doi: 10.1371/journal.pone.0208082 (PMC6294362; doi:10.1371/journal.pone.0208082)
Supplement: S1 Appendix — We implemented a Gibbs sampler algorithm to draw from the posterior distribution of the proposed model. We adopt a parameter expansion strategy to cope with high correlations among the parameters. (PDF) [file pone.0208082.s001.pdf]

S1 Appendix. Parameter expansion. We implemented a Gibbs sampler algorithm to draw from the posterior distribution of the proposed model. We adopt a parameter expansion strategy to cope with high correlations among the parameters.

Specifically, instead of assuming $\boldsymbol{w}_n = \boldsymbol{w} + \boldsymbol{v}_n$, we assume

$$\boldsymbol{w}_n = \boldsymbol{w} + \boldsymbol{\alpha} * \boldsymbol{v}_n \tag{1}$$

where $\boldsymbol{\alpha}$ are vector-valued variable of length $R^2 p$, assumed to follow multivariate Normal distributions

$$\boldsymbol{\alpha} \sim \text{MVN}(\mathbf{0}, aI) \tag{2}$$

where a is a constant and $*$ stands for element-wise multiplication. In this specification, the true patient-level coefficients become

$$\boldsymbol{v}_n^* = \boldsymbol{\alpha} * \boldsymbol{v}_n \tag{3}$$

and the true precision of these coefficients become

$$\theta_{vk}^* = \alpha_k^{-2} \theta_{vk} \tag{4}$$

for $k = 1, \dots, R^2 p$, where α_k is the k-th elements of $\boldsymbol{\alpha}$. Note that we are only interested in the posterior inference of $\boldsymbol{v}_n^*$ and θ_{vk}^*.
