# Supplementary material for: Bayesian hierarchical vector autoregressive models for patient-level predictive modeling
Source: PLoS One. 2018 Dec 14;13(12):e0208082. doi: 10.1371/journal.pone.0208082 (PMC6294362; doi:10.1371/journal.pone.0208082)
Supplement: S2 Appendix — To draw from posterior distribution using Gibbs sampler, one draws each parameter from its conditional distribution given all other parameters at their current values. We provide full conditionals for all parameters. (PDF) [file pone.0208082.s002.pdf]

S2 Appendix. Full conditional distributions. To draw from posterior distribution using Gibbs sampler, one draws each parameter from its conditional distribution given all other parameters at their current values. We provide full conditionals for all parameters.

Let $g(\cdot)$ denote conditional distribution of one parameter given others. We now give the full conditional of all parameters.

1. $g(\boldsymbol{w}) = \text{MVN}\left(M_w, S_w^2\right)$, where

$$S_w^2 = \left[\sum_{n=1}^{N}\left(H_n H_n^T\right) \otimes \Lambda + D\right]^{-1}$$

$$M_w = S_w^2 \left[\sum_{n=1}^{N}\left(H_n H_n^T\right) \otimes \Lambda\left(\hat{\boldsymbol{w}}_n - \boldsymbol{\alpha} * \boldsymbol{v}_n\right)\right]$$

where $\hat{\boldsymbol{w}}_n$ is the MLE of the VAR coefficients for the n-th patient.

2. $g(\boldsymbol{v}_n) = \text{MVN}\left(M_{v_n}, S_{v_n}^2\right), n = 1, \ldots, N$, where

$$S_{v_n}^2 = \left[\text{Diag}(\boldsymbol{\alpha})\left(\left(H_n H_n^T\right) \otimes \Lambda\right)\text{Diag}(\boldsymbol{\alpha}) + \Theta_v\right]^{-1}$$

$$M_{v_n} = S_{v_n}^2\left[\text{Diag}(\boldsymbol{\alpha})\left(\left(H_n H_n^T\right) \otimes \Lambda\right)\left(\hat{\boldsymbol{w}}_n - \boldsymbol{w}\right)\right]$$

where $\text{Diag}(\cdot)$ represents assigning the $R^2 p$ vector on a diagonal matrix.

3. $g(\boldsymbol{\alpha}) = \text{MVN}\left(M_\alpha, S_\alpha^2\right)$, where

$$S_\alpha^2 = \left[\sum_{n=1}^{N}\text{Diag}(\boldsymbol{v}_n)\left(\left(H_n H_n^T\right) \otimes \Lambda\right)\text{Diag}(\boldsymbol{v}_n) + aI\right]^{-1}$$

$$M_\alpha = S_\alpha^2\left[\sum_{n=1}^{N}\text{Diag}(\boldsymbol{v}_n)\left(\left(H_n H_n^T\right) \otimes \Lambda\right)\left(\hat{\boldsymbol{w}}_n - \boldsymbol{w}\right)\right]$$

4. $g(\theta_{vk}) = \Gamma(k_v, s_{vk}), k = 1, \ldots, R^2 p$, where

$$k_v = \frac{N}{2} + k$$

$$s_{vk} = \left(\frac{1}{2}\sum_{n=1}^{N} v_{nk}^2 + \frac{1}{s}\right)^{-1}$$

where v_{nk} is the k-th element of $\boldsymbol{v}_n$.

5. $g(\lambda_{1,k}^2) = \Gamma\left(\mu_{\lambda_{1,k}^2}, \nu_{\lambda_{1,k}^2}\right), k = 1, \ldots, R^2 p$, where

$$\nu_{\lambda_{1,k}^2} = \nu_1 + 2$$

$$\mu_{\lambda_{1,k}^2} = \frac{(\nu_1 + 2)(\mu_1 \xi_k^2)}{\nu_1 \xi_k^2 + 2\tau_k^2 \mu_1}$$

6. $g(\lambda_{2,k}) = \Gamma\left(\mu_{\lambda_{2,k}}, \nu_{\lambda_{2,k}}\right), k = 1, ..., B^2 p$, where

$$\nu_{\lambda_{2,k}} = \nu_2 + 1$$

$$\mu_{\lambda_{2,k}} = \frac{(\nu_2+1)\mu_2}{\nu_2 + w_k^2 \mu_2}$$

where w_k is the k-th element of $\boldsymbol{w}$.

7. $g(\Lambda) = \text{Wishart}(S_\Lambda, \nu_\Lambda)$, where

$$\nu_\Lambda = \sum_{n=1}^{N} \left(T_n - p\right) + \nu + 2Rp$$

$$S_\Lambda^{-1} = \left[\sum_{n=1}^{N} S_n + K^{-1} + 2QQ^T\right]^{-1}$$

where

$$S_n = (Y_n - W_n H_n)(Y_n - W_n H_n)^T$$

is the residual matrix of the n-th patient,

$$Y_n = \begin{pmatrix} \boldsymbol{y_{n,p+1}} & \cdots & \boldsymbol{y_{n,T_n}} \end{pmatrix}$$

is a $R \times (T_n - p)$ matrix,

$$W_n = vec^{-1}(\boldsymbol{w} + \boldsymbol{\alpha} * \boldsymbol{v}_n)$$

where $vec^{-1}(\cdot)$ means stacking the $R^2 p$-coefficient vector back into a $R \times Rp$ matrix, and

$$Q = \begin{pmatrix} \gamma_1 & \cdots & \gamma_{(Rp-1)R+1} \\ \vdots & \ddots & \vdots \\ \gamma_R & \cdots & \gamma_{R^2 p} \end{pmatrix}$$

is a $R \times Rp$ matrix composed of $\gamma_k, k = 1, ..., R^2 p$, which depend on $2\tau_k^2, k = 1, ..., R^2 p$ and Λ through

$$\tau_{R^2 p} \lambda_{1,R^2 p} = \gamma_{R^2 p}$$

$$\tau_k \lambda_{1,k} = \gamma_k + \boldsymbol{m}_k \left(\gamma_{k+1}, ..., \gamma_{B^2 p}\right)^T$$

for $k = 1, ..., R^2 p - 1$, where

$$\boldsymbol{m}_k = \left(M_{k,k+1}, ..., M_{k,R^2 p}\right) \begin{pmatrix} M_{k+1,k+1} & \cdots & M_{k+1,R^2 p} \\ \vdots & \ddots & \vdots \\ M_{R^2 p,k+1} & \cdots & M_{R^2 p,R^2 p} \end{pmatrix}^{-1}$$

8. $g(\frac{1}{2\tau_k^2}) = \sqrt{\frac{b_k}{2\pi}} \left(\frac{1}{2\tau_k^2}\right)^{-\frac{3}{2}} exp\left\{-\frac{b_k\left(\frac{1}{2\tau_k^2} - a_k\right)^2}{2a_k^2 \frac{1}{2\tau_k^2}}\right\}, k = 1, ..., R^2 p$, where

$$a_k = \sqrt{\frac{\lambda_{1,k}^2}{w_k^2 \xi_k^2}}$$

$$b_k = \frac{\lambda_{1,k}^2}{\xi_k^2}$$

Note that the conditional distribution of $2\tau_k^2$ is complicated, but Park and Casella (2008) have shown that $g(\frac{1}{2\tau_k^2})$ is Inverse-Gaussian distribution. Therefore, we draw $\frac{1}{2\tau_k^2}$ first from $g(\frac{1}{2\tau_k^2})$ and then transform back to get $2\tau_k^2$.
