# Supplementary figures and images for: Bayesian hierarchical vector autoregressive models for patient-level predictive modeling
Source: PLoS One. 2018 Dec 14;13(12):e0208082. doi: 10.1371/journal.pone.0208082 (PMC6294362; doi:10.1371/journal.pone.0208082)

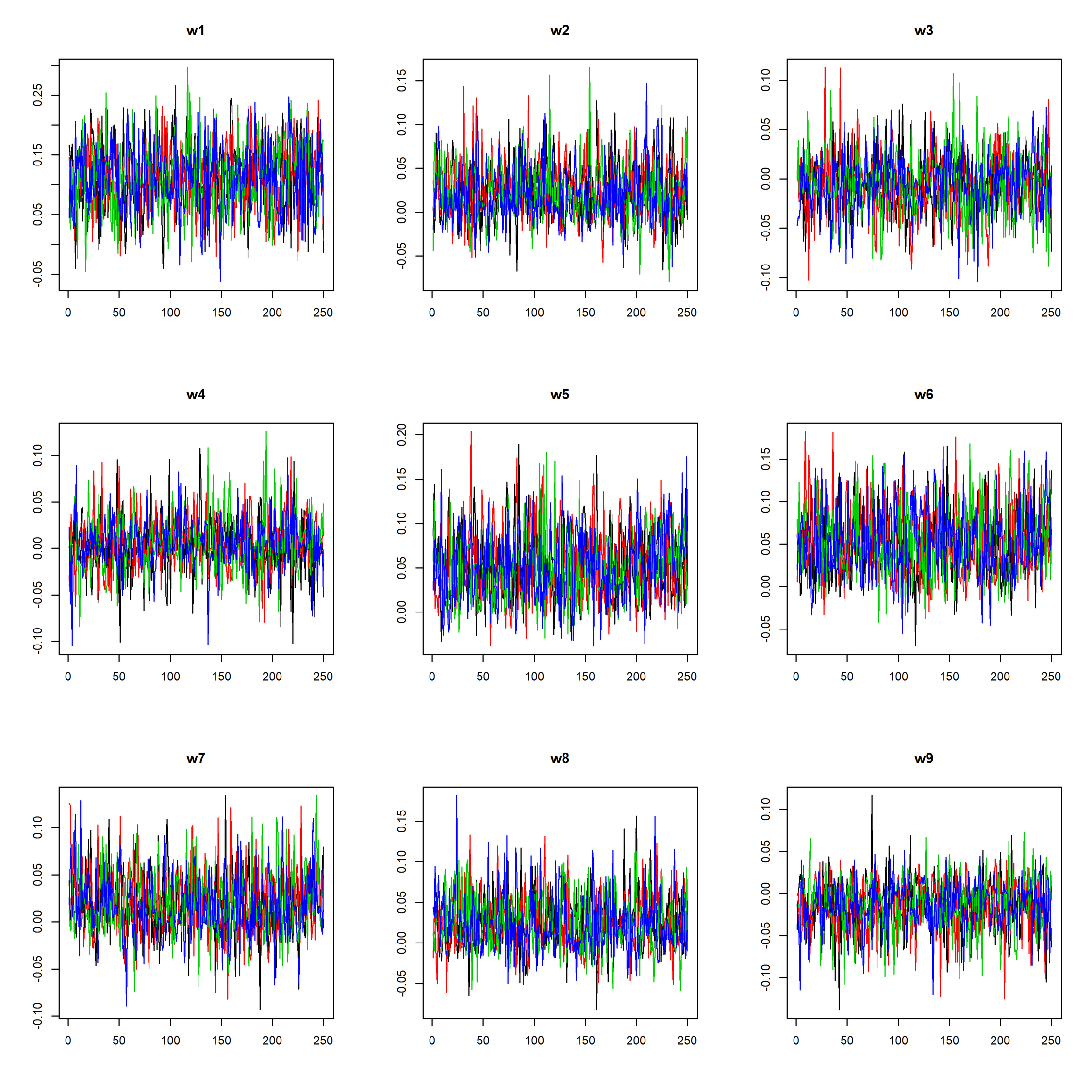

Supplement: S1 Fig — w1 to w9 indicate the 9 population-level coefficients. The 4 chains are displayed by different colors in each panel. (TIF) [file pone.0208082.s003.tif]

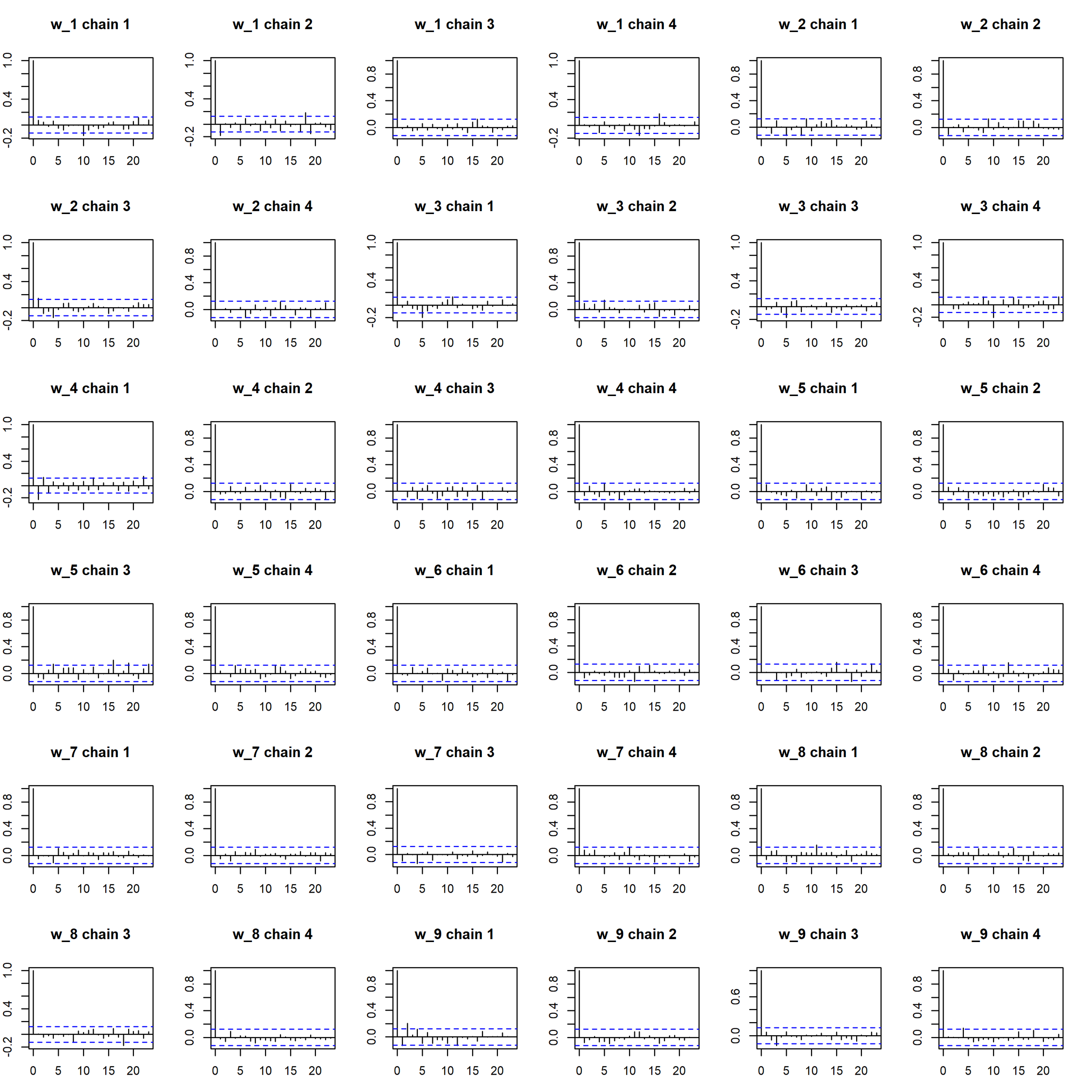

Supplement: S2 Fig — w1 to w9 indicate the 9 population-level coefficients. The 4 chains are displayed by different colors in each panel. (TIF) [file pone.0208082.s004.tif]

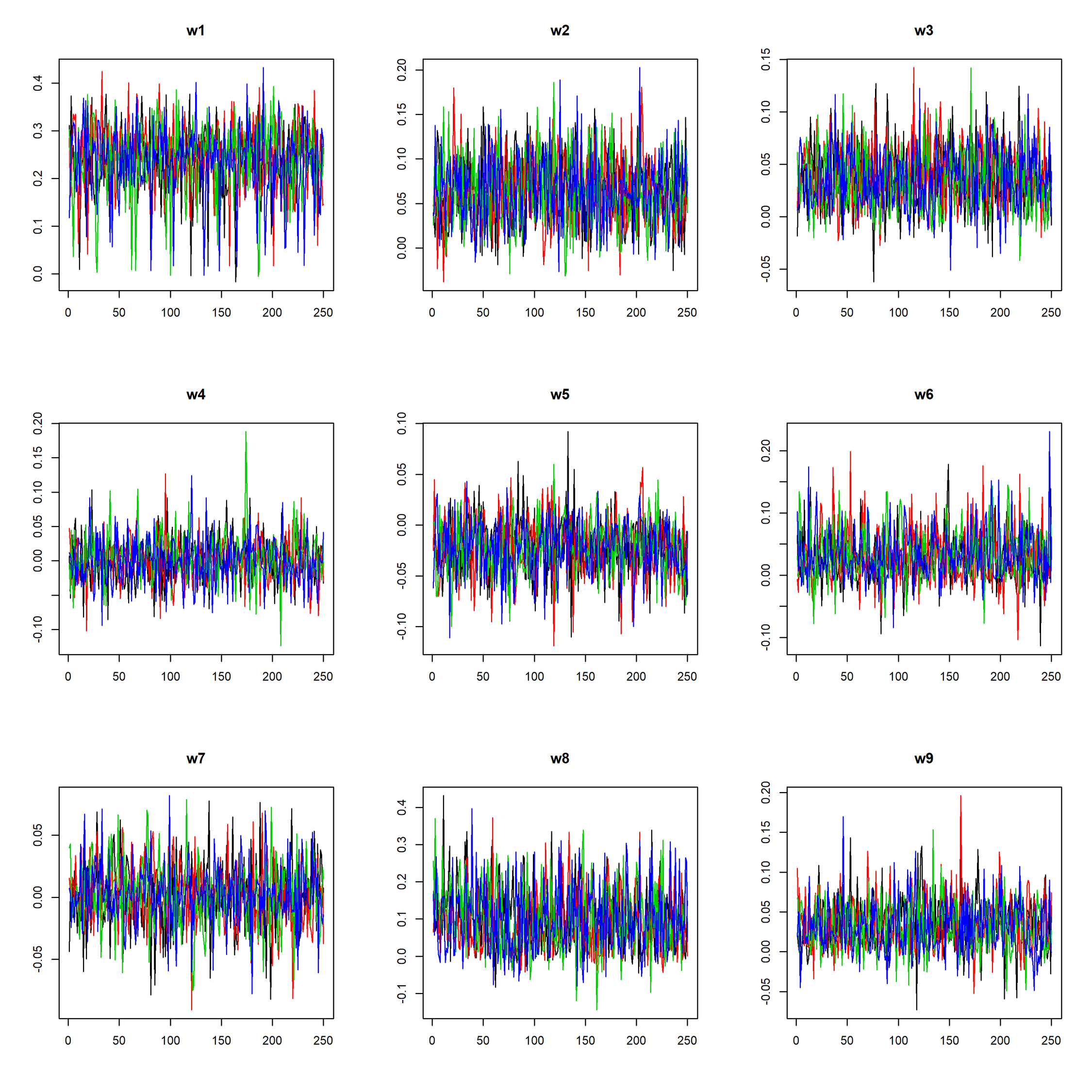

Supplement: S3 Fig — w1 to w9 indicate the first 9 population-level coefficients. The 4 chains are displayed by different colors in each panel. (TIF) [file pone.0208082.s005.tif]

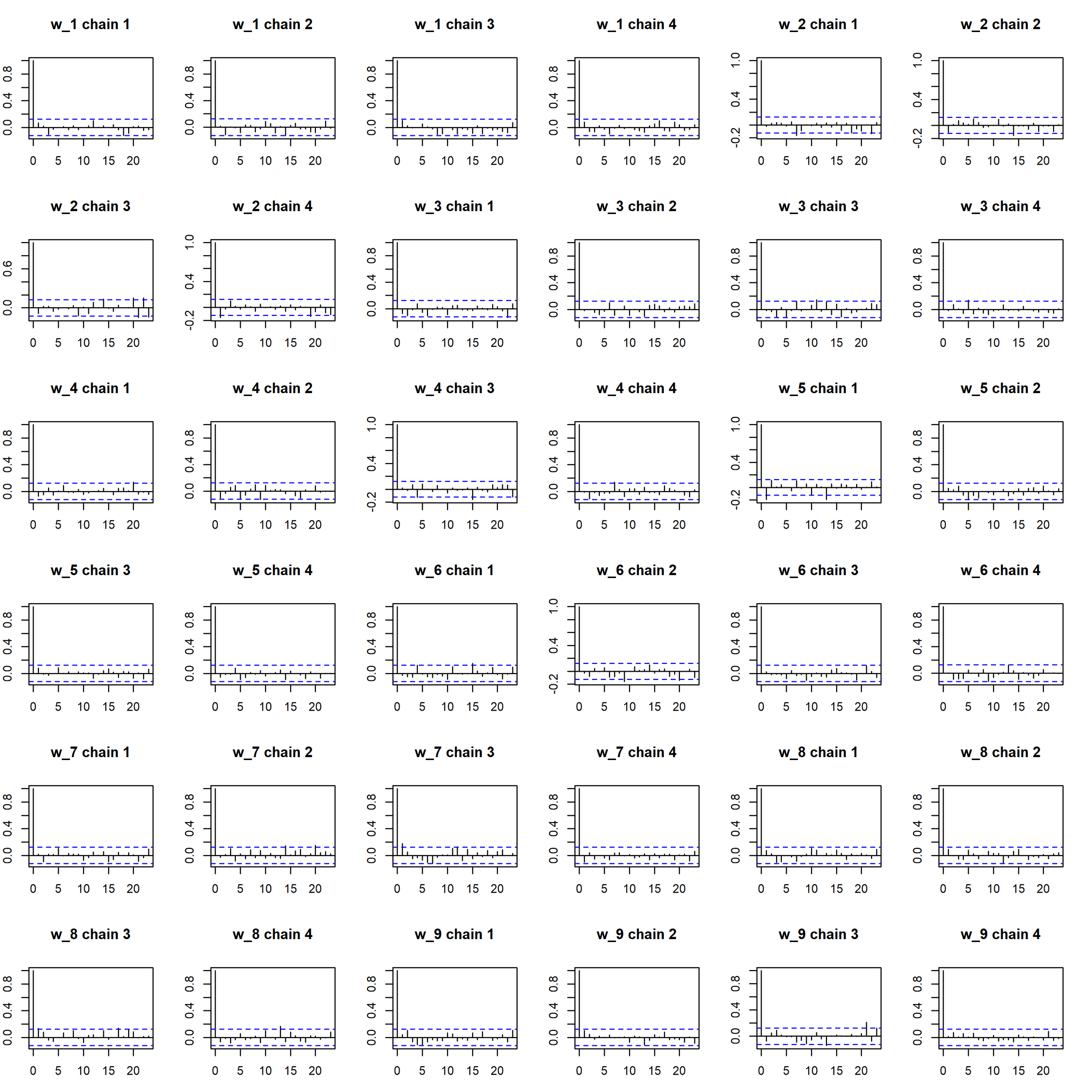

Supplement: S4 Fig — w1 to w9 indicate the 9 population-level coefficients. The 4 chains are displayed by different colors in each panel. (TIF) [file pone.0208082.s006.tif]

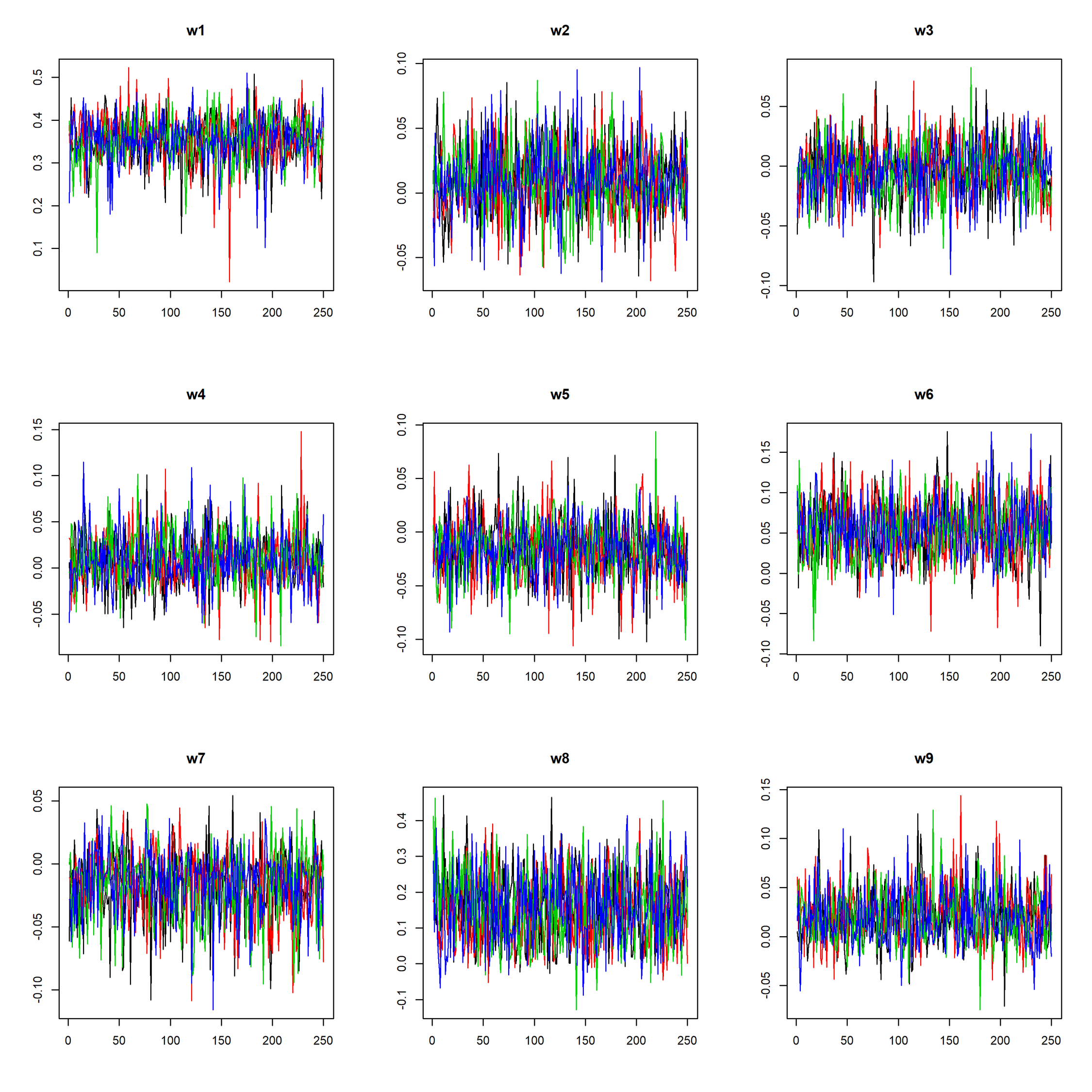

Supplement: S5 Fig — w1 to w9 indicate the first 9 population-level coefficients. The 4 chains are displayed by different colors in each panel. (TIF) [file pone.0208082.s007.tif]

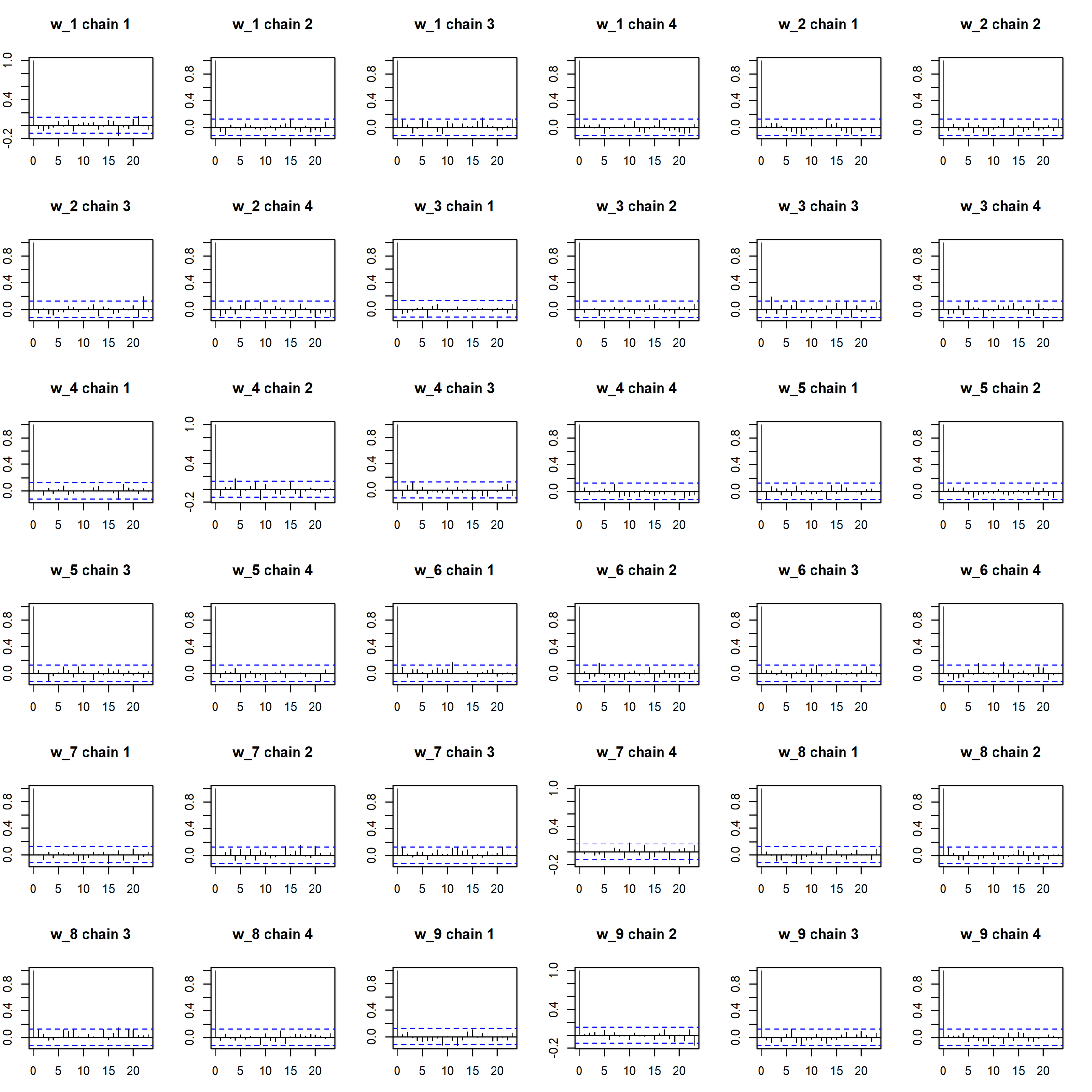

Supplement: S6 Fig — w1 to w9 indicate the 9 population-level coefficients. The 4 chains are displayed by different colors in each panel. (TIF) [file pone.0208082.s008.tif]

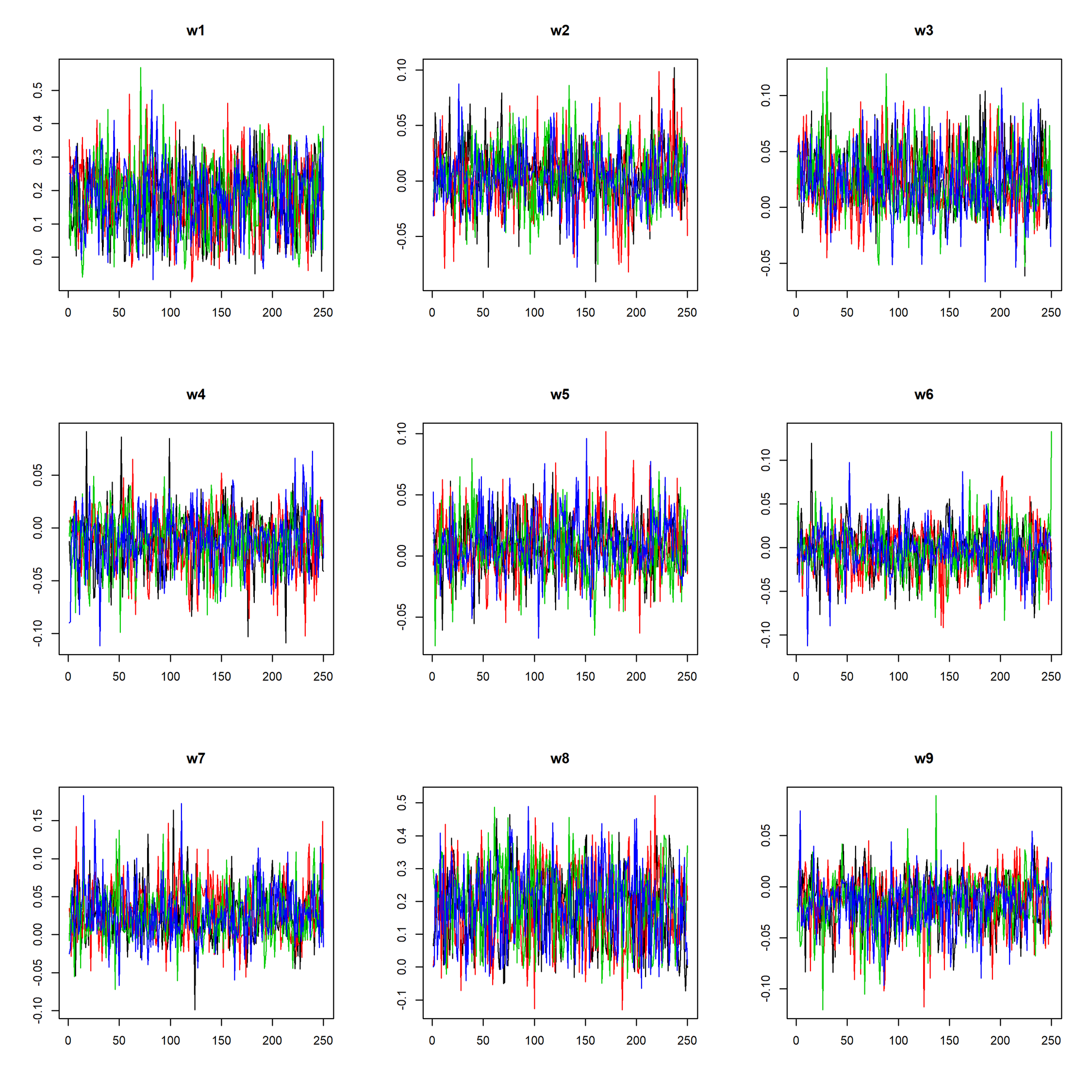

Supplement: S7 Fig — w1 to w9 indicate the first 9 population-level coefficients. The 4 chains are displayed by different colors in each panel. (TIF) [file pone.0208082.s009.tif]

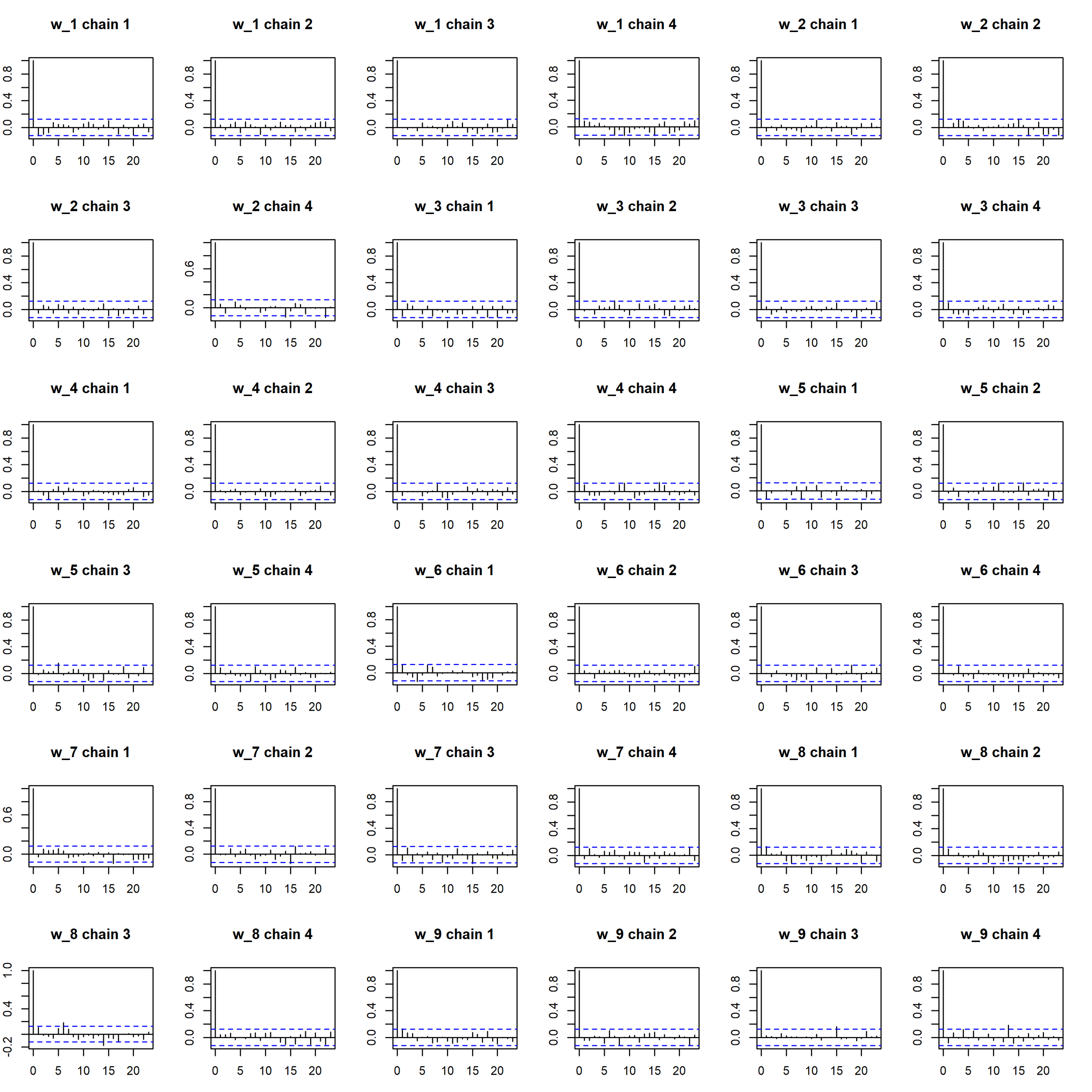

Supplement: S8 Fig — w1 to w9 indicate the 9 population-level coefficients. The 4 chains are displayed by different colors in each panel. (TIF) [file pone.0208082.s010.tif]

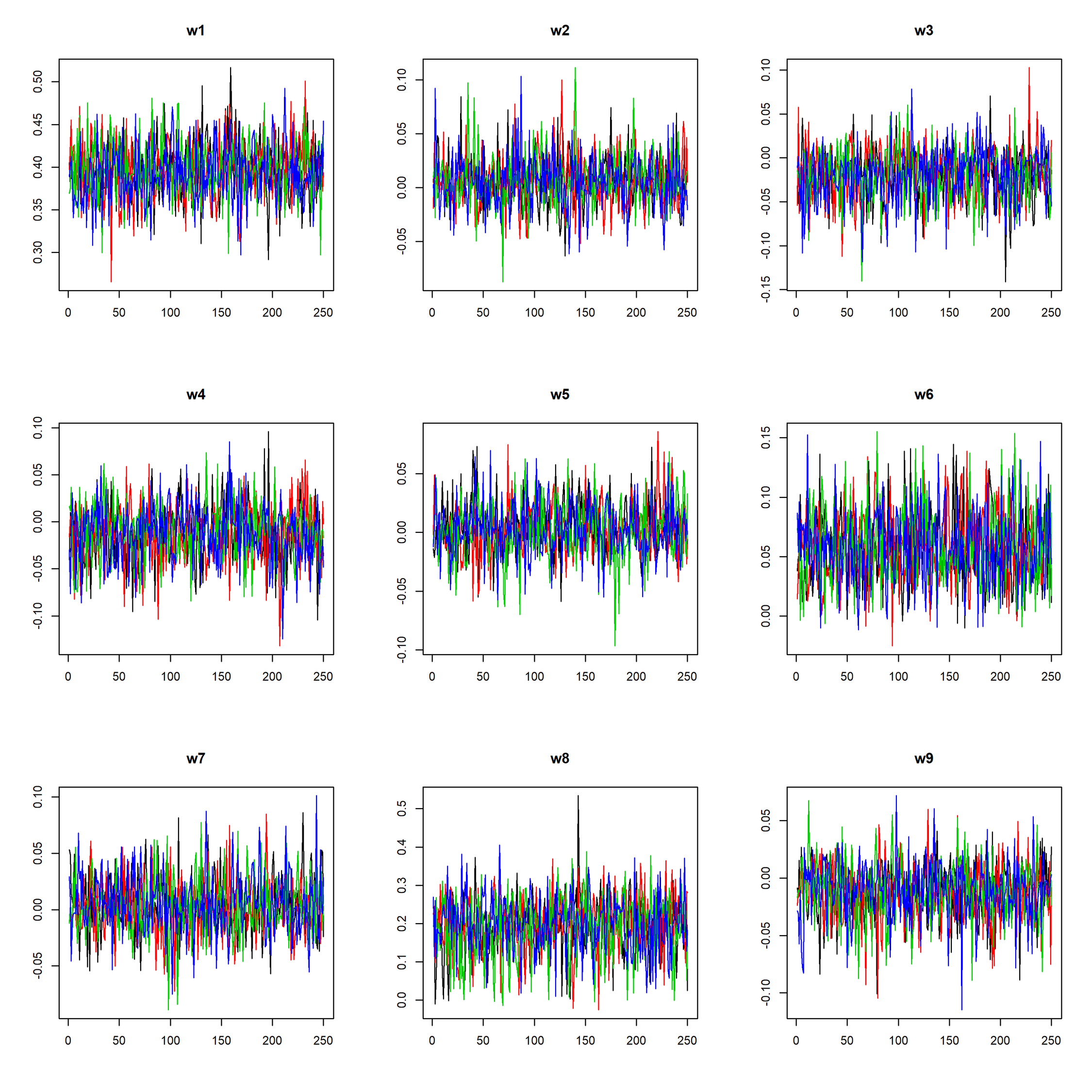

Supplement: S9 Fig — w1 to w9 indicate the first 9 population-level coefficients. The 4 chains are displayed by different colors in each panel. (TIF) [file pone.0208082.s011.tif]

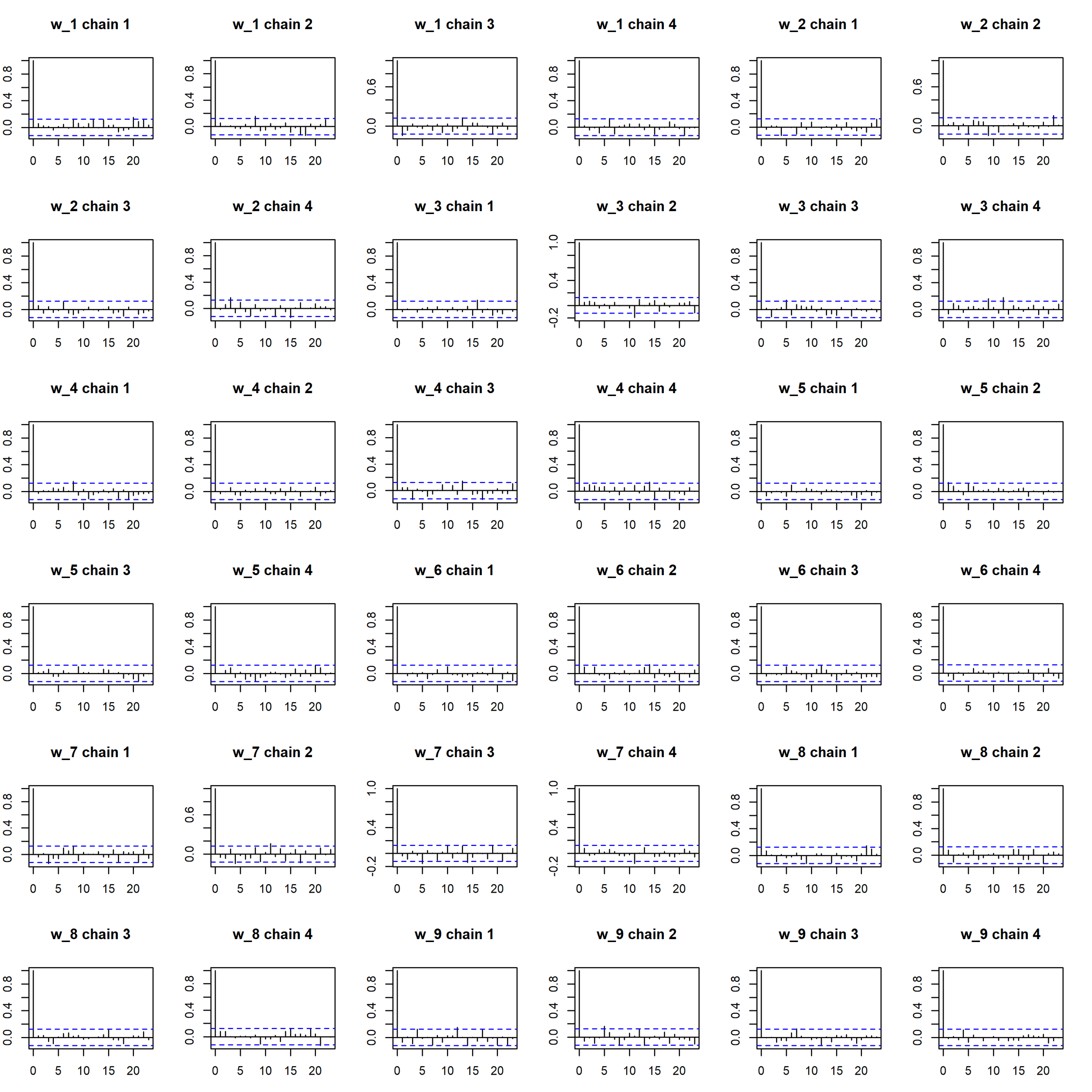

Supplement: S10 Fig — w1 to w9 indicate the 9 population-level coefficients. The 4 chains are displayed by different colors in each panel. (TIF) [file pone.0208082.s012.tif]
