# Supplementary material for: Bayesian hierarchical vector autoregressive models for patient-level predictive modeling
Source: PLoS One. 2018 Dec 14;13(12):e0208082. doi: 10.1371/journal.pone.0208082 (PMC6294362; doi:10.1371/journal.pone.0208082)
Supplement: S1 Table — Each row is for one patient and each column is for one VAR coefficient. (PDF) [file pone.0208082.s013.pdf]

S1 Table. Patient-level coefficients obtained by the Bayesian VAR models. Each row is for one patient and each column is for one VAR coefficient.

ID	T=>T	T=>N	T=>C	N=>T	N=>N	N=>C	C=>T	C=>N	C=>C
1	-0.009	0	0.002	0	0	0	-0.003	0.005	0
2	0.009	-0.001	0	0.001	-0.001	0	0.006	0	0
3	0.054	0	0	0.001	0	-0.001	-0.002	0.003	0
4	-0.092	-0.001	-0.001	0	0	0	0.015	0.002	0.001
5	-0.006	0	0.001	0.001	-0.001	0	-0.008	-0.001	-0.001
6	-0.003	0	0	0.001	0	0	0.017	-0.011	0
7	-0.131	0	-0.001	0	0	0	0.007	-0.002	0
8	0.001	0.001	-0.003	0	0	0	-0.002	-0.003	0
9	0.022	0.001	0	0	0	0.001	0.005	0.005	0
10	0.013	0	0	0	0	-0.001	-0.002	0.003	0
11	-0.028	0.004	-0.001	0	0	0	0.003	0.002	-0.001
12	-0.016	-0.001	0	0	0	0	-0.021	0.004	0
13	0.011	0	0.001	0	0	0.001	0	-0.009	0
14	-0.004	0.001	-0.001	0	0	0.001	-0.02	0	0
15	0.001	0	0	0.001	0	0.001	0.003	0.012	0
16	0.014	0	0	0.001	0	0	-0.02	0.001	-0.001
17	-0.019	-0.004	0.003	0	0	0	0.021	0.006	-0.001
18	-0.026	0	0.001	0	0	0	0.001	0	0.001
19	0.388	0.001	-0.001	0	0	0	0.011	0.003	-0.001
20	0.228	0.002	-0.001	0	-0.001	0	0.011	0	0
21	-0.004	-0.001	0.001	0.001	0.001	0	0.007	-0.007	0
22	0.018	-0.002	-0.001	0.001	0	0	0.009	-0.001	0
23	0.012	0.001	0	0	0	0	-0.001	0.006	0
24	-0.004	0	-0.001	0	0	0	0.007	-0.001	0
25	0.018	0	-0.001	0	0	0	0.002	-0.007	0

T=tobacco use, N=negative affect, C=craving; “X=>Y” represents the lag-1 association of variable X in the previous day on Y in the current day.
