# Supplementary material for: Bayesian hierarchical vector autoregressive models for patient-level predictive modeling
Source: PLoS One. 2018 Dec 14;13(12):e0208082. doi: 10.1371/journal.pone.0208082 (PMC6294362; doi:10.1371/journal.pone.0208082)
Supplement: S2 Table — Each row is for one patient and each column is for one VAR coefficient. (PDF) [file pone.0208082.s014.pdf]

S2 Table. Patient-level coefficients obtained by the patient-specific VAR model. Each row is for one patient and each column is for one VAR coefficient.

ID	T=>T	T=>N	T=>C	N=>T	N=>N	N=>C	C=>T	C=>N	C=>C
1	-0.124	-0.024	0.307	-0.819	0.075	-0.258	0.32	0.118	-0.051
2	0.035	-0.392	-0.315	0.002	-0.347	-0.639	0.17	0.081	0.134
3	0.51	-0.337	-0.593	-0.155	0.08	-0.161	-0.04	0.05	-0.097
4	-0.218	-0.007	-0.127	0.042	-0.195	-0.291	0.296	0.176	0.291
5	-0.144	0.283	0.014	0.085	-0.194	0.107	-0.311	-0.208	-0.285
6	-0.08	0.239	0.398	0.061	-0.104	0.177	0.276	-0.593	-0.345
7	-0.466	-0.013	-0.343	0.102	0.266	0.217	0.266	-0.1	0.561
8	-0.012	-0.327	-0.591	0.046	0.474	0.21	-0.108	-0.249	-0.138
9	-0.04	0.208	0.124	-0.194	0.445	0.165	0.172	-0.064	-0.026
10	-0.048	0.093	-0.148	-0.005	-0.126	-0.212	-0.158	0.235	0.048
11	-0.384	0.568	-0.212	-0.217	0.157	-0.05	0.296	0.002	0.242
12	-0.234	-0.15	0.173	0.036	-0.01	0.147	-0.512	0.128	-0.154
13	0.169	-0.111	0.123	0.146	0.177	0.378	-0.243	-0.432	-0.097
14	0.359	-0.378	1.407	0.088	-1.003	0.885	-0.841	0.854	-0.415
15	-0.178	0.069	-0.004	0.322	0.518	0.354	-0.003	0.365	-0.018
16	0.151	0.216	0.26	0.26	0.162	-0.138	-0.653	-0.287	-0.096
17	-0.125	-0.398	0.258	-0.082	0.069	-0.01	0.36	0.125	-0.048
18	-0.44	0.268	0.011	0.248	-0.163	-0.078	-0.218	0.243	0.298
19	0.737	0.136	-0.128	-0.279	0.027	0.047	0.173	-0.048	-0.318
20	0.432	0.141	-0.251	-0.012	0.086	-0.018	0.408	-0.141	0.397
21	-0.006	-0.01	0.186	0.078	0.036	-0.164	-0.008	-0.334	-0.156
22	0.055	-0.295	-0.21	0.018	0.027	-0.125	0.15	-0.039	0.059
23	0.072	0.237	0.141	-0.01	-0.397	-0.434	-0.088	0.405	0.074
24	-0.053	0.056	-0.231	0.226	-0.172	0.162	0.23	0.048	0.031
25	0.034	-0.073	-0.249	0.013	0.114	-0.271	0.067	-0.335	0.106

T=tobacco use, N=negative affect, C=craving; “X=>Y” represents the lag-1 association of variable X in the previous day on Y in the current day.
