# Supplementary material for: Bayesian hierarchical vector autoregressive models for patient-level predictive modeling
Source: PLoS One. 2018 Dec 14;13(12):e0208082. doi: 10.1371/journal.pone.0208082 (PMC6294362; doi:10.1371/journal.pone.0208082)
Supplement: S3 Table — Each row is for one patient and each column is for one VAR coefficient. (PDF) [file pone.0208082.s015.pdf]

S3 Table. Patient-level coefficients obtained by the regularized linear regression model. Each row is for one patient and each column is for one VAR coefficient.

ID	T=>T	T=>N	T=>C	N=>T	N=>N	N=>C	C=>T	C=>N	C=>C
1	0	-0.319	0	0	0	0	0	0	0
2	0	0	0	0	0	0	0	0	0
3	0.255	0	0	0	0	0	0	0	0
4	0	0	0	0	0	0	0	0	0
5	0	0	0	0	0	0	0	0	0
6	0	0	0	0	0	0	0	0	0
7	0	0	0	0	0	0	0	0.054	0.144
8	0	0	0	0	0	0	0	0	0
9	0	0	0	0	0	0	0	0	0
10	0	0	0	0	0	0	0	0	0
11	0	0	0	0	0	0	0	0	0
12	0	0	0	0	0	0	0	0	0
13	0	0	0	0	0	0	0	0	0
14	0	0	0	0	0	0	0	0	0
15	0	0	0	0	0.319	0.194	0	0	0
16	0	0	0	0	0	0	0	0	0
17	0	0	0	0	0	0	0	0	0
18	0	0	0	0	0	0	0	0	0
19	0.527	0	0	0	0	0	0	0	0
20	0.33	0	0.144	0	0	0	0	0	0
21	0	0	0	0	0	0	0	0	0
22	0	0	0	0	0	0	0	0	0
23	0	0	0	0	0	0	0	0	0
24	0	0	0	0	0	0	0	0	0
25	0	0	0	0	0	0	0	0	0

T=tobacco use, N=negative affect, C=craving; "X=>Y" represents the lag-1 association of variable X in the previous day on Y in the current day.
